# Dynamics of the Herpes simplex virus DNA polymerase holoenzyme during DNA synthesis and proof-reading revealed by Cryo-EM

**DOI:** 10.1093/nar/gkae374

**Published:** 2024-05-29

**Authors:** Emil Gustavsson, Kay Grünewald, Per Elias, B Martin Hällberg

**Affiliations:** Department of Cell and Molecular Biology, Karolinska Institutet, 171 77 Stockholm, Sweden; Centre for Structural Systems Biology, Deutsches Elektronen-Synchrotron DESY, Notkestraße 85, Building 15, 22607 Hamburg, Germany; Centre for Structural Systems Biology, Deutsches Elektronen-Synchrotron DESY, Notkestraße 85, Building 15, 22607 Hamburg, Germany; Leibniz-Institute of Virology, Martinistraße 52, 20251 Hamburg, Germany; Department of Chemistry, University of Hamburg, Martin-Luther-King-Platz 6, 20146 Hamburg, Germany; Institute of Biomedicine, Department of Medical Biochemistry and Cell Biology, Sahlgrenska Academy, University of Gothenburg, Box 440, 405 30 Gothenburg, Sweden; Department of Cell and Molecular Biology, Karolinska Institutet, 171 77 Stockholm, Sweden; Centre for Structural Systems Biology, Deutsches Elektronen-Synchrotron DESY, Notkestraße 85, Building 15, 22607 Hamburg, Germany

## Abstract

Herpes simplex virus 1 (HSV-1), a double-stranded DNA virus, replicates using seven essential proteins encoded by its genome. Among these, the UL30 DNA polymerase, complexed with the UL42 processivity factor, orchestrates leading and lagging strand replication of the 152 kb viral genome. UL30 polymerase is a prime target for antiviral therapy, and resistance to current drugs can arise in immunocompromised individuals. Using electron cryo-microscopy (cryo-EM), we unveil the dynamic changes of the UL30/UL42 complex with DNA in three distinct states. First, a pre-translocation state with an open fingers domain ready for nucleotide incorporation. Second, a halted elongation state where the fingers close, trapping dATP in the dNTP pocket. Third, a DNA-editing state involving significant conformational changes to allow DNA realignment for exonuclease activity. Additionally, the flexible UL30 C-terminal domain interacts with UL42, forming an extended positively charged surface binding to DNA, thereby enhancing processive synthesis. These findings highlight substantial structural shifts in the polymerase and its DNA interactions during replication, offering insights for future antiviral drug development.

## Graphical Abstract

**Graphical Abstract:**
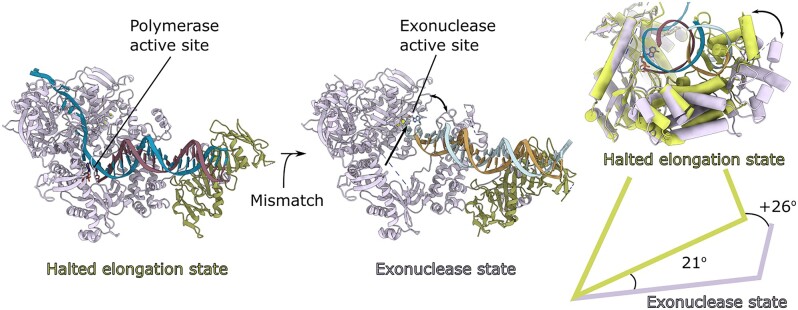

## Introduction

Herpesviruses are large dsDNA viruses infecting metazoans ranging from molluscs to humans. In vertebrates, they exist in three sub-families referred to as alpha, beta and gamma herpesviruses. Herpesviruses cause productive or lytic infections, causing a wide range of symptoms in the infected host, but they can also enter a life-long latent state from which new infectious cycles can reactivate. Herpesviruses are highly prevalent in the adult population, but virus replication is under efficient control by the immune system. In immunocompromised individuals, however, the infections become much more severe, and the development of resistance to antiviral compounds occurs more frequently 1–4). Eight different herpesviruses can infect human beings. Herpes simplex viruses 1 and 2 (HSV-1 and HSV-2, respectively), also called human herpes virus 1 and 2 (HHV1 and HHV2, respectively), and varicella-zoster virus (VZV or HHV3), are alphaherpesviruses replicating in epithelial cells and they enter the latent state in sensory neurons (5). HSV-1 and HSV-2 commonly cause oral or genital cold sores. Recurrent infections affecting the eye may result in visual loss. In rare instances, HSV-1 may also give rise to devastating encephalitis (6–8). The infections may be treated using acyclovir, which is a guanosine analogue phosphorylated by a virus-specific thymidine kinase to become a chain-terminating inhibitor of viral DNA synthesis (9). Human cytomegalovirus (HCMV or HHV5) and human herpesvirus 6 and 7 (HHV6 and HHV7) are betaherpesviruses. HCMV causes mononucleosis, the roseolavirus HHV6, and, to a lesser extent, HHV7 is associated with benign exanthema subitum and sometimes opportunistic infections in immunocompromised individuals (10). The gammaherpesviruses Epstein-Barr virus (EBV or HHV4) and human herpesvirus 8 (HHV8) cause infectious mononucleosis, nasopharyngeal carcinoma and lymphoma (EBV), as well as Kaposi's sarcoma (HHV8) (11). More recently, EBV has become a prime candidate for the initiation of multiple sclerosis (12). Antiviral therapies exist for some of these viruses, but new and more efficient drugs are needed.

Despite the large variation in the resulting diseases, the herpesviruses all share a common DNA replication cycle, and the replication apparatus, including DNA polymerase, is largely conserved (13). Initiation of HSV-1 DNA replication occurs at the origins of replication activated by the origin-binding protein encoded by gene UL9 (14,15). The HSV-1 DNA polymerase, encoded by the UL30 gene, forms a 190kDa heterodimeric complex with its processivity factor UL42 protein (16,17). They are part of a six-component molecular machine together with the helicase-primase complex, encoded by the UL5, UL8 and UL52 genes, and the UL29 single-strand DNA-binding protein often referred to as ICP8 (13,18). This molecular machine, the replisome, can be reconstituted *in vitro*, and it is capable of carrying out coordinated synthesis of leading and lagging strands on a primed minicircle template (19,20). While functionally dependent on species-specific protein-protein interactions, the replisome is highly conserved amongst all herpesviruses infecting vertebrates.

The HSV-1 UL30 is a family B DNA polymerase containing a 5′-3′ polymerase activity and a proofreading 3′-5′ exonuclease activity. The UL30 DNA polymerase has the characteristic fold of many polymerases (21–24), and although there is little sequence conservation, the UL42 protein is structurally similar to a single PCNA subunit but does not form a ring-like clamp (25). UL42, which binds directly to DNA, instead interacts as a monomer with the extreme C-terminus of UL30 (26) and promotes processive DNA synthesis (16,17). The heterodimeric complex forms a highly processive DNA polymerase in HSV-1, which can complete DNA synthesis on a primed single-stranded circle *in vitro* (27). Previous crystallographic studies of UL30 and UL42 have been able to determine the apo-structure of UL30 (21) or together with an inhibitor and DNA (22) and of UL42 bound to a small peptide consisting of 35 residues from the UL30 C-terminus (25).

As part of an effort to present high-resolution structural models of HSV-1 DNA replication in various functional states, we here report the structure of the UL30/UL42 heterodimeric complex bound to primed template DNA using cryo-EM. The complex is captured in three states: (i) a pre-translocation state with the primer terminus in the pocket for the incoming dNTP, (ii) a halted elongation state with a dideoxycytidinemonophosphate (ddCMP) at the primer terminus and dATP in the nucleotide-binding pocket, and (iii) a DNA-editing state with one or two mismatched base pairs in the primer-terminus at the 3′-5′ exonuclease active site.

Our results show that significant structural changes occur in the DNA polymerase itself and in its interaction with DNA during different stages of DNA synthesis. Furthermore, we show how the UL42 protein binds to the flexible C-terminus of DNA polymerase. We also observe a positively charged surface in the holoenzyme, which helps to explain how processive DNA replication is achieved.

## Materials and methods

### Construction of expression vectors

The HSV-1 UL30 and UL42 genes (gene-ID: 2703462 and 24271471, respectively) were codon optimized for expression in insect cells and ordered as gene fragments with an N-terminal Strep-tag from Genscript. NEBuilder HiFi DNA Assembly Cloning Kit (NEB, cat. #E5520S) was used to assemble the gene fragments into a pFastBac plasmid without additional affinity tags (Gibco, cat. #10360014). The sequence-verified pFastBac plasmid containing gene UL30 or UL42 was transformed into DH10Bac cells (Gibco, cat. #10361012), and bacmid incorporation was analysed with two rounds of blue-white screening and PCR of resulting bacmids.

### Protein expression and purification

The Gibco ExpiSf Expression System (cat. #A38841) from ThermoFisher Scientific was used for the development of recombinant baculovirus and protein expression. ExpiSf9 cells were transfected with recombinant bacmid, and P0 baculovirus stock was harvested 96 h post-transfection. P0 baculovirus stocks were subsequently employed to co-infect ExpiSf9 cells for simultaneous expression of the DNA polymerase and its processivity factor. After 48 h, cells were harvested and lysed using a lysis buffer (20 mM HEPES pH 7.8, 400 mM NaCl, 1.5 mM MgCl_2_, 1 mM DTT, 5% glycerol (v/v), 1× cOmplete protease inhibitor cocktail (Roche, cat. #11697498001), 1 μg/ml DNase 1 (ITW reagents, cat. #A3778) and 0.1% (v/v) Triton X-100 (ITW reagents, cat. #142314)) employing a Dounce homogenizer. The resulting lysate was clarified through two rounds of centrifugation (20 min at 15 000×g followed by 40 min at 25 000×g). The supernatant was filtered through a 0.2 μm syringe filter and loaded onto a StrepTrap HP column (Cytiva, cat. #28907547) pre-equilibrated with an equilibration buffer (20 mM HEPES pH 7.8, 400 mM NaCl, 1.5 mM MgCl_2_, 1 mM DTT, 5% glycerol (v/v)). Protein was eluted by adding 10 mM desthiobiotin (IBA Life Sciences, cat. #2-1000-002). The purity of the protein was evaluated through SDS-PAGE analysis.

### Proof-reading reaction

DNA oligonucleotides were from Integrated DNA Technologies and used to demonstrate that the proteins used in our structural studies were enzymatically active. First, a DNA template strand was annealed to a primer strand with a mismatched nucleotide at the 3′-terminus, as highlighted in bold below, in 10 mM HEPES pH 7.8, 100 mM NaCl in a 1:1 molar ratio. The mixture was heated to 95°C and allowed to cool slowly to room temperature. The buffer in our protein samples was adjusted to 20 mM HEPES pH 7.8, 150 mM NaCl, 5 mM MgCl_2_ and 2 mM DTT and the protein solutions were concentrated to 2 μM. DNA synthesis was carried out at a protein:DNA molar ratio of 1:50 and dNTPs at a concentration of 2 mM. The reaction was incubated at 37°C for 3 h. The DNA was purified with the NucleoSpin Gel and PCR Clean-up kit (Macherey-Nagel, cat. #740609.50) and sent for sequencing using a strand-specific primer for newly synthesised DNA. The results are shown in Supplementary Table S2 with the mismatched nucleotide in the primer at position 39.

DNA template strand: 5′-TGTAAAACGACGGCCAGTTAGATCGATCGATCGATATTTGCTGACCTTTGTTCTAATTGAGTTGGTTGGACGGCTGCGAGGCGATCAAGGTGTCGTAGTG-3′ DNA primer strand: 5′-CACTACGACACCTTGATCGCCTCGCAGCCGT**A**-3′ Sequencing primer: 5′-TGTAAAACGACGGCCAGT-3′

### *In-vitro* reconstitution of protein complexes for cryo-EM

In general, the *in-vitro* reconstitution of all samples followed the same procedure, with specific variations outlined below. DNA oligonucleotides were procured from Integrated DNA Technologies. Protein buffer was exchanged to 20 mM HEPES pH 7.8, 150 mM NaCl, 5 mM MgCl_2_ and 2 mM DTT and the protein was concentrated to 2 μM in a Vivaspin 500 centrifugal concentrator with a 10 000 MWCO (Sartorius, cat. #VS0102). Primed DNA was added in a protein:DNA molar ratio of 1:2. The reaction was incubated at 37°C for 30 min, and aliquots of 3.5 μl were then applied to glow-discharged UltrAufoil R1.2/1.3 Au 300 mesh grids (Quantifoil), immediately blotted for 2.5 s and plunged into liquid ethane using an FEI Vitrobot IV (4°C, 95% relative humidity).

### Exonuclease state DNA polymerase

The protein buffer contained 5 mM CaCl_2_ instead of MgCl_2_. The primed DNA had a 1- or 2-bp mismatch (marked in bold) and phosphorothioate bonds marked with an asterisk in the sequences below:

2 base pair mismatch DNA template strand: 5′-ATTTGCTGACCTTTGTTCTGG**GG**TGAGTTGGTTGGACGGCTGCGAGGCGATCAAGGTGTCGTAGTGGC-3′

2 base pair mismatch DNA primer strand: 5′-GCCACTACGACACCTTGATCGCCTCGCAGCCGTCCAACCAACTCA*A*A-3′

1 base pair mismatch DNA template strand: 5′-ATTTGCTGACCTTTGTTCTGG**G**TGAGTTGGTTGGACGGCTGCGAGGCGATCAAGGTGTCGTAGTGGC-3′

1 base pair mismatch DNA primer strand:

5′-GCCACTACGACACCTTGATCGCCTCGCAGCCGTCCAACCAACTC*A*A-3′

### Halted elongation state DNA polymerase

DNA template strand: 5′-ATTTGCTGACCTTT**G**TTCTAATTGAGTTGGTTGGACGGCTGCGAGGCGATCAAGGTGTCGTAGTGGC-3′

DNA primer strand: 5′-GCCACTACGACACCTTGATCGCCTCGCAGCCGTCCAACCAACTCA-3′, together with a 1 mM mix of dATP, dGTP, dTTP and **di**deoxy CTP (**d**dCTP) to halt the polymerization at the first guanosine (marked in bold).

### Pre-translocation state DNA polymerase

DNA template strand: 5′-CGAAAGTACGTTATTGCGACTGGCCGTCGCTCTACAACGTCGTGACTG-3′

DNA primer strand: 5′-CAGTCACGACGTTGTAGAGCGA-3′.

### Electron cryomicroscopy

For data collection, we used the following instrumentation and workflow.

### Halted elongation DNA polymerase dataset

A Krios G3i electron microscope at the Centre for Structural Systems Biology (CSSB) Cryo-EM facility, operated at an accelerating voltage of 300 kV equipped with a K3 BioQuantum (Gatan) was used for collecting the halted elongation state DNA polymerase data. Cryo-EM data were acquired using EPU software (Thermo Fisher) at a nominal magnification of 105kX, with a pixel size of 0.85 Å per pixel. Movies of a total fluence of ∼ 50 electrons/Å^2^ were collected at ∼ 1 e^−^/Å^2^ per frame. A total number of 2484 movies were acquired at an underfocus range of 0.5–2.0 μm.

### Exonuclease and pre-translocation state DNA polymerase datasets

A Krios G3i electron microscope at the Karolinska Institute (KI) 3D-EM facility, operated at an accelerating voltage of 300 kV equipped with E-CFEG or X-FEG, and a K3 BioQuantum was used for collecting exonuclease (E-CFEG) and pre-translocation state DNA polymerase data (X-FEG). Cryo-EM data were acquired using EPU software (FEI) at a nominal magnification of 165 kX, with a pixel size of 0.505 Å per pixel (pixel size for 1-bp mismatch exonuclease state DNA polymerase was 0.508). Movies of a total fluence of ∼58 electrons/Å^2^ were collected at ∼0.7 e^−^/Å^2^ per frame. A total number of 8467 (pre-translocation state DNA polymerase); 12 820 (2-bp mismatch exonuclease state DNA polymerase); 25 892 (1-bp mismatch exonuclease state DNA polymerase) movies were acquired at an underfocus range of 0.3–1.5 μm.

### Cryo-EM image processing

Motion correction was performed with RELION’s own implementation of MotionCor2 (28) and CTF estimation with CTFFind 4.1 (29). Particles were picked using Warp 1.0.9 (30) and extracted in RELION 4.0b (31). The particles were then imported to cryoSPARC v.3.2.0 (32). One round of 2D classification was performed for each dataset, and the subsequent refinement strategies for each dataset can be seen below.

### Halted elongation and pre-translocation state DNA polymerase dataset

Following 2D classification, ab-initio reconstruction was performed with three or four classes for pre-translocation and halted elongation states, respectively. One round of heterogeneous refinement was performed together with an additional ‘noise class’ reconstructed from 10 000 particles excluded during an initial 2D classification. This was followed by non-uniform refinement. The volume from non-uniform refinement was used together with all imported particles in two new rounds of heterogeneous refinement to recover good particles initially excluded in the 2D classification. Another round of non-uniform refinement was performed. The resulting particles were exported to RELION for Bayesian polishing and CTF refinement (the pre-translocation state data was subjected to two additional rounds of 3D classification in RELION focused on the active site). The particles were then re-imported into cryoSPARC for one final non-uniform refinement followed by local refinement focusing on UL30 and UL42, respectively. Representative processing workflows for pre-translocation and halted elongation states can be seen in Supplementary Figures S1 and S2, respectively.

### 2-bp mismatch exonuclease state DNA polymerase dataset

After 2D classification, Ab-initio reconstruction with 1 class was conducted, along with three ‘noise classes’ constructed from 30 000 excluded particles in a subset. Three rounds of heterogeneous refinement were performed and the output volume was used to template pick the full dataset. Four additional rounds of heterogeneous refinement were performed, and the particles were exported for 3D classification in RELION. The resulting particles were re-imported to cryoSPARC for non-uniform refinement. Global and local CTF refinement was performed followed by a reconstruction and local refinement focusing on UL30 and UL42, respectively. From these particles, further subclassifications were performed, focusing on the exonuclease active site and the ssDNA plus β-hairpin loop. cryoSPARC v.4.1 and 3D flexible refinement was also used to improve the flexible regions, e.g. ssDNA plus β-hairpin loop. A representative processing workflow can be seen in Supplementary Figure S3.

### 1-bp mismatch exonuclease state DNA polymerase dataset

After 2D classification, Ab-initio reconstruction with three classes was conducted, along with three ‘noise classes’ constructed from 100 000 excluded particles in a subset. Three rounds of heterogeneous refinement were performed, and output volume was used together with all imported particles in three new rounds of heterogeneous refinement. Another round of non-uniform refinement was performed. The resulting particles were subjected to global and local CTF refinement, a 3D classification without image alignment, followed by homogenous reconstruction. Focused classification and refinement were performed for UL30 and UL42, respectively. A representative workflow can be seen in Supplementary Figure S4.

### Post-processing

The half maps from the local refinement jobs were merged in ChimeraX v.1.3 (33) and post-processed with LocSpiral (34), DeepEMhancer (35), or by local resolution estimation and local filtering in cryoSPARC (32).

### Model building

The initial model building involved fitting PDB-ID: 7LUF (crystal structure of inhibited UL30 (22)) and 1DML (crystal structure of UL42 (25)) into the density map for the pre-translocation state UL30/UL42 complex using Coot v.0.9.8 (36). Iterative model building was performed in Coot, followed by Ramachandran and rotamer fitting in ISOLDE v.1.3 (37) and refinement with Servalcat (38). In lower resolution areas, models were cross-referenced against each other and the crystal structures for enhanced consistency and model completeness (Data collection parameters, refinement and validation statistics can be found in Supplementary Table S1).

## Results and discussion

### Overall structure of the HSV-1 UL30/UL42 ternary complex

Here, we examined by cryo-EM the UL30 DNA polymerase in complex with DNA, nucleotide and the processivity factor UL42 in three functionally relevant states referred to as the pre-translocation state, the halted elongation state, and the DNA-editing state.

**Figure 1.**
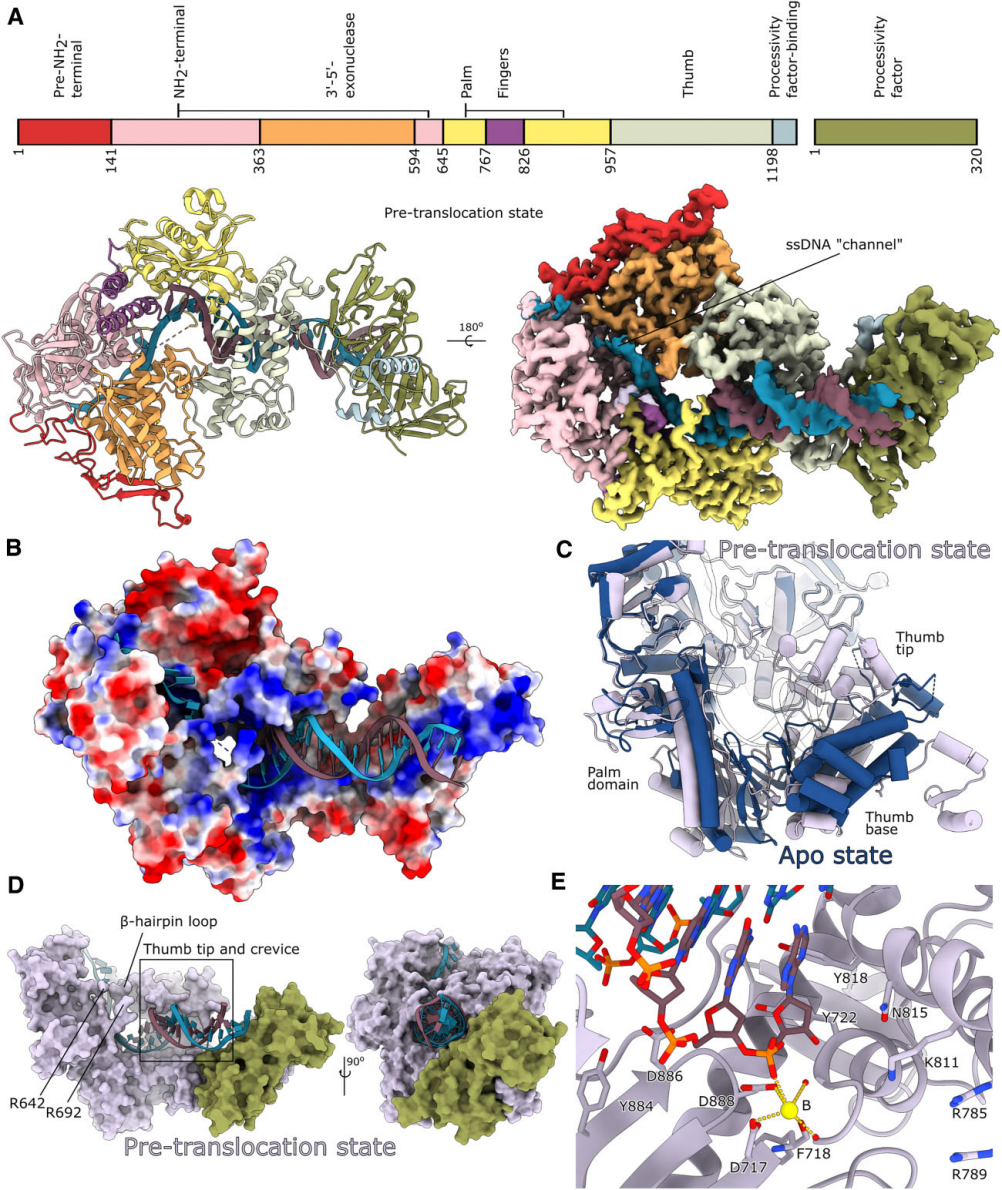
(**A**) Structure and cryo-EM map (postprocessed with LocSpiral (34)) of pre-translocation state UL30, UL42 and DNA, coloured by domains. The fold of UL30 follows the general principle of B family DNA polymerase domains: Pre-NH_2_-terminal (red, residues 1–140), NH_2_-terminal (pink, residues 141–362 and 594–700), 3′-5′ exonuclease (orange, residues 363–593), Palm (yellow, residues 645–766 and 826–956), Fingers (purple, residues 767–825), Thumb (sage, residues 957–1197) and UL42-binding domain (light blue, residues 1198–1235). The UL42 processivity factor is coloured moss green. Single-stranded template DNA density can extend between the NH_2_-terminal and exonuclease domains in the so-called ‘ssDNA-channel’. (**B**) A molecular surface representation of the UL30-UL42 complex with bound DNA, coloured by the local electrostatic potential (blue, +8 kT/e; red, 5 kT/e). (**C**) Pre-translocation state structure of UL30 (plum color) compared to a previously solved crystal structure of apo-UL30 (dark blue) (PDB-ID: 2GV9) (21). The apo-structure thumb domain is in a relatively more open conformation, while the palm domain is flexing in towards the active site. These changes could be due to crystal-packing artefacts. (**D**) The β-hairpin loop forms a clasp with residues 640–642 and 691–698, and the unstructured region 643–690 is likely involved as well. The tip of the thumb domain interacts with both the minor and major groove of the double-stranded DNA. UL42 (moss green) does not alter its structure upon binding to DNA but the contact occurs only through DNA phosphate–UL42 side chain interactions. (**E**) Polymerase active site in the pre-translocation state. The 3′-end of the primer strand is located in the seat of the incoming dNTP, while the fingers domain is in the open conformation.

The UL30 DNA polymerase revealed a classic family B DNA polymerase architecture. This architecture comprises fingers, thumb, and palm domains flanked by an NH_2_-terminal domain (NTD) and an exonuclease domain (Figure 1A). In the holo-enzyme complex, the UL30 C-terminal hooks into UL42, and UL30/UL42 forms an extended positively charged surface bound by the primer-template phosphate backbone (Figure 1B). Notably, the interaction between UL30 and UL42 exhibits remarkable flexibility, and the primer-template DNA can exhibit dynamic bending facilitated by UL42 acting as a tether (Supplementary Movies S1 and S2).

A previous structure of the apo-enzyme determined by crystallography resembles, in general, the pre-translocation state determined by cryo-EM in this report (see superimposition in Figure 1C) (21). One notable exception is that in our structure, the thumb and NH_2_-terminal domains are farther apart; the thumb adopts a more open conformation, and the palm domain is closer to the polymerase active site. Such a difference could be an artefact caused by crystal packing but, nevertheless, reflects a capacity for the polymerase to adapt to the presence of DNA (Figure 1C). Furthermore, the pre-translocation state adopts an overall fold similar to a previous ternary enzyme complex crystal structure (22), with the fingers domain in an open conformation. Due to the inhibitor present in the crystal structure of the ternary enzyme complex, the primer strand was in the post-translocation state.

A less well-resolved density was observed for single-stranded DNA, extending within a ‘ssDNA-channel’ extending between the NH_2_-terminal and 3′-5′ exonuclease domains (Figure 1A). In gp43 from RB69 an additional cleft was identified, and it was proposed to bind the template DNA (39). Liu *et al.* suggested that this cleft would serve a similar purpose in UL30 (21). However, neither of our structures shows the template extending into this cleft, and we find no evidence for a bound nucleotide. In fact, in UL30, several bulky side chains prevent nucleotide binding in this region.

### The structure of the HSV-1 UL30/UL42 pre-translocation state

We first captured a pre-translocation state complex displaying an overall resolution of 2.4 Å (Figure 1A, Supplementary Figure S5). In the structure, UL30 and UL42 together cover 24 bp of double-stranded primer-template DNA, and they are located on one side of the double helix. The DNA polymerase can, therefore, freely bind to and dissociate from double-stranded DNA without undergoing major conformational changes. However, several key structural features in the DNA polymerase help to position DNA correctly. The β-hairpin loop consisting of residues 500–516, which has previously been implicated as a control mechanism to orient the DNA strands correctly in gp43 (40–42), forms a clasp holding the single-stranded template strand in place. Residues 640–642 and 691–698 form the second part of this clasp. It also seems likely that parts of the unstructured region in between (residues 643–690) are further involved (Figure 1D). Furthermore, the thumb domain interacts with the double-stranded DNA’s minor and major groove, promoting correct positioning and rotation of the DNA. Curiously, while UL30 reorganizes to facilitate multiple interactions with the DNA, forming a crevice in which the DNA slides, UL42 does not change shape compared to the crystal structure of the DNA-free form (Figure 1D, Supplementary Figure S6) (25).

Notably, the active site of DNA polymerase in our pre-translocation state is occupied by the 3′-terminal nucleotide in the position where an incoming dNTP would bind (Figure 1E). The fingers domain exhibits an open conformation, primed for dNTP binding, in a manner consistent with the Brownian-ratchet model. This model posits toggling between pre- and post-translocated states until a new dNTP occupies the pocket. The lowest energy state would be the pre-translocated state, and this state would, therefore, constitute the majority of the population. A small subpopulation of the post-translocated state should also exist (43). However, we were unable to identify this sub-population in which the dNTP pocket still should be empty. Possibly, an incoming nucleotide is needed to facilitate translocation in the UL30 DNA polymerase. This would contrast with the general view that translocation has to occur before a new dNTP can move in (44).

### Structure of the HSV-1 UL30/UL42 halted DNA polymerase state

**Figure 2.**
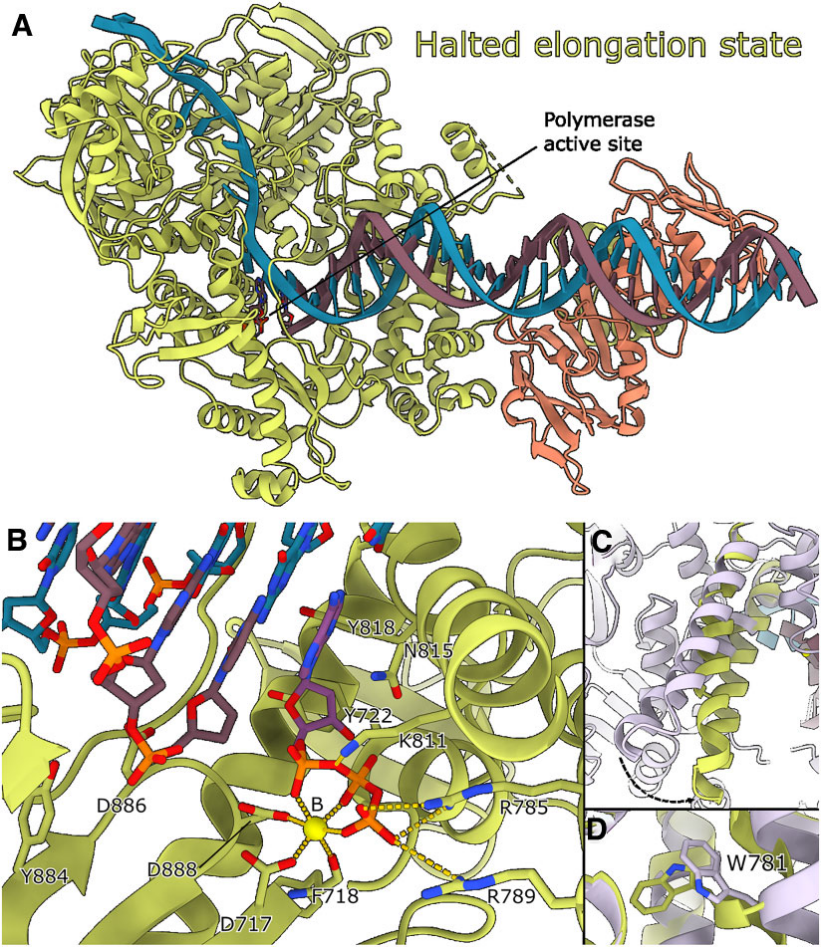
(**A**) The ternary structure of UL30/UL42 in the halted elongation state. (**B**) The polymerase active site of the halted elongation state structure. The coordination of side chains is very similar to previous predictions obtained from homology modelling of gp43 from RB69 by Liu et al. and Zarrouk *et al.* (21,24). D888, D717 and the main chain carbonyl of F718, together with the three phosphate groups in dATP coordinate metal B. The fingers domain has now closed, and residues R785 and R789 in the fingers domain are in range to interact with the terminal phosphate group. Metal B is slightly shifted compared to its location in the pre-translocation state structure (Figure 1E). (**C**) Upon binding of dATP, the fingers domain moves from open in the pre-translocation state (plum colour) to closed in the halted-elongation state (marked in green). (**D**) W781, which has been implicated in sensitivity towards foscarnet, acyclovir and ganciclovir, flips upon closing of the fingers domain and appears to act as an equilibrium regulator. Mutation W781V leads to an 11-fold decreased polymerase activity and higher EC50 values for antiviral drugs (46).

Furthermore, we captured a halted-elongation state at an overall resolution of 2.5 Å. Here, we introduced a **d**dCTP after an 8-bp elongation (Figure 2A, Supplementary Figure S7). The incoming dATP resided within the polymerase pocket, and the fingers domain was observed to be in a closed conformation (Figure 2B and C). However, in the absence of a hydroxyl group at the 3′ end of ddCTP, the primer cannot be further elongated. In the active site of the halted elongation state structure the incoming dATP is well-resolved together with the Mg^2+^ ion, metal B, which is associated with catalysis (23). The second Mg^2+^ ion, metal A, was not resolved in the absence of the coordinating 3′-hydroxyl group in ddCTP (Figure 2B). We identified a density corresponding to Mg^2+^ in the exonuclease site, as also observed in the pre-translocation structure.

Our findings corroborate and expand upon the insights from Liu et al. (21) and Zarrouk *et al.* (24), who employed homology modelling with gp43 from RB69 to predict active site interactions. Our study demonstrates the direct involvement of highly conserved residues D717, D888 and the main-chain carbonyl group of F718 in coordinating the metal ion. Furthermore, residues R785, R789 and K811 coordinate the phosphate groups of dATP. Additionally, N815 and Y818 coordinate the dATP, while Y884 and D886 help coordinate the primer strand. Y722 could potentially interfere with the binding of ribonucleotides due to steric clashes with the extra 2′ hydroxyl group and work as a ‘steric gate’ (21,45). Intriguingly, a specific tryptophan residue (W781) located in the fingers domain exhibited a conformational change upon closing (Figure 2D). This residue, implicated in resistance to antiviral drugs upon mutation (46), appears to act as an equilibrium regulator. Notably, the W781V mutation led to an 11-fold decreased polymerase activity and higher EC50 values for antiviral drugs (46).

### The structure of the HSV-1 UL30/UL42 DNA editing state

**Figure 3.**
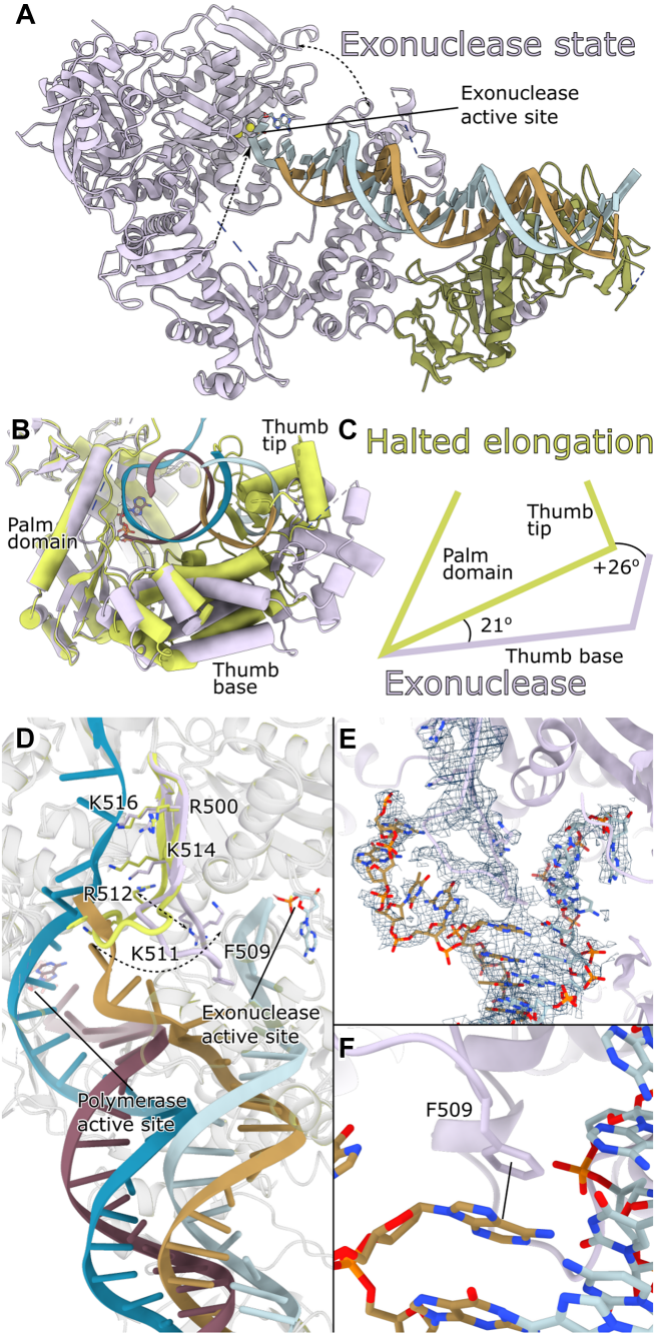
(**A**) The ternary structure of UL30/UL42 in the editing state (2-bp mismatch structure). (**B**) Halted elongation (green) and 2-bp mismatch exonuclease (plum) state structures have been overlaid based on the palm domain, and the relative movements of the thumb domain are shown. The thumb domain consists of two subdomains, the ‘thumb base’ and ‘thumb tip’. (**C**) Schematic representation of the relative movements, where the thumb base rotates by 21° relative to the palm domain. The thumb tip rotates an additional 26° relative to the thumb base. (**D**) The β-hairpin loop shifts along with the movement of the DNA from the polymerase active site to the exonuclease site. The outer loop (residues 503–513) rotates to allow for K511 and R512 to interact with the primer strand in the exonuclease site. In contrast, the inner loop β-strands with residues R500, K514 and K516 remain oriented towards the ssDNA-channel to interact with the template strand. F509 stacks with the DNA base in the template strand. (**E**) Subclassification of the particle set revealed a class where the density is better refined, and we could, from this class, model part of the template strand as well as the complete β-hairpin loop (density only shown around the β-hairpin loop and DNA for clarity). (**F**) The conserved residue F509 stacks with a DNA base in the template strand to stabilize the separation of strands in the exonuclease state.

We expanded our investigation by capturing the UL30/UL42 complex in a DNA editing state with the primer terminus in the active site of the 3′-5′ exonuclease at an exceptional overall resolution of 1.9 Å (Figure 3A, Supplementary Figure S8 and Supplementary Figure S9). In this state, we introduced either a 1-bp or 2-bp mismatch accompanied by a phosphorothioate bond and replaced Mg^2+^ with Ca^2+^ to halt exonuclease activity. A 2-bp mismatch has been shown to be the optimal substrate for exonuclease activity (47). Of the three states examined here, the exonuclease state complex reached the highest resolution with an overall resolution of 1.9Å (2-bp mismatch structure, Supplementary Figures S8 and S10). The core of UL30 is best resolved, and for the more peripheral processivity factor, the resolution is 2.3Å. With the movement of the DNA to the exonuclease state, the entire polymerase core ‘opens up’. The thumb and exonuclease domains move apart, allowing for the DNA to shift between active sites. In addition, residues 1030–1075 also move relative to the thumb domain in general as an extra joint (Figure 3B). The domain rotation in UL30, as measured by ChimeraX (33), is larger with a 21° rotation for the thumb base relative to the palm domain and an additional 26° for the tip relative to the thumb base (Figure 3C) but still keeping the distance between the polymerase and exonuclease active sites constant (40Å).

The single-stranded template DNA extending in the ssDNA-channel between the NH_2_-terminal and exonuclease domains visible in the pre-translocation state and halted elongation state structures (Figure 1A) is not entirely resolved in either of the exonuclease state structures. In fact, there seems to be quite a lot of flexibility in this state, as the DNA bases located just outside the active site exhibit poor resolution. Subsequent classification, focusing on the single-stranded template DNA, indicated that the template strand remained oriented towards this ssDNA channel, as seen in the pre-translocation state and halted elongation state structures. However, even within this sub-classification, the single-stranded DNA within the actual ssDNA channel remained relatively poorly defined (Figure 3D and E).

The β-hairpin loop is also poorly resolved but has become repositioned with the DNA primer strand. The β-strands maintain their relative positions, facilitating the orientation of R500, K514, and K516 towards the ssDNA channel and enabling interactions with the template strand. The outer loop part, residues 503–513, has rotated along with the DNA molecule, and K511 and R512 now seem to interact with the primer strand (Figure 3D). The conserved F509 also positions itself to stack with the DNA base in the template strand and stabilizes the separation of the DNA strands (Figure 3F). The β-hairpin loop position is well conserved between the two different exonuclease structures, strengthening our assignment.

To conclude, our structures provide further evidence for a mechanism in which the β-hairpin loop is involved in the separation of the DNA strands and the coordination of the two active sites.

### The HSV-1 UL30/UL42 exonuclease active site in its editing state

**Figure 4.**
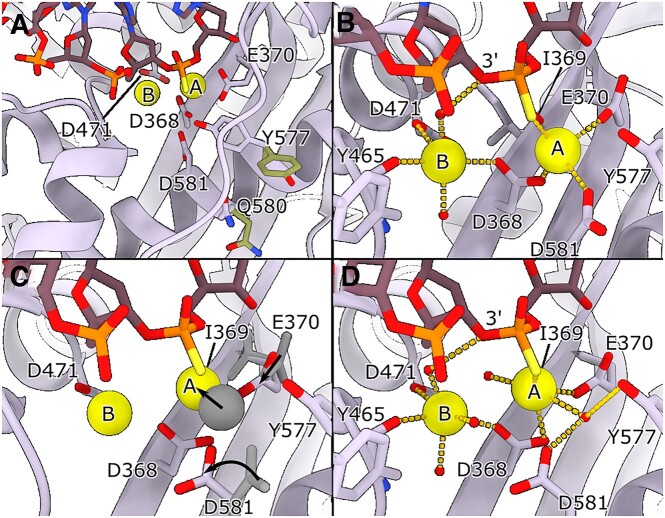
(**A**) The exonuclease active site of the 2-bp mismatch exonuclease state structure. We show the densities for two Ca^2+^ ions in the active site. We used Ca^2+^ and a nucleotide with a phosphorothioate bond to inhibit exonuclease activity. Double conformations for both Y577 and Q580 (colored in green and plum) were observed in the exonuclease site. Y577 orients both towards the active site and away from it. (B–D) Further classification of the particles makes it possible to distinguish two different sub-states.(**B**) A less populated and lower resolution sub-state with a slightly different coordination. Metal B is coordinated by D368, Y465 and D471 as well as two water molecules. D368, E370, D581 and the thiophosphate group in the primer strand of DNA coordinate metal A. (**C**) A comparison between the two sub-states in panel B and D. Metal A shifts position closer to I369 ‘underneath’ the thiophosphate. The side chains of E370 and D581 subsequently rotate in. (**D**) The highest resolution sub-state of the active site. D368, D471, the backbone of Y465 and three water molecules coordinate metal B. One of the water molecules also interacts with the bridging 3′-oxygen. The water molecule missing in panel B is in position to interact with metal B. Metal A is coordinated by D368, the main chain carbonyl of I369, E370, two water molecules and the thiophosphate group of the primer strand of DNA. One of the water molecules also interacts with Y577 and D581. This water molecule, likely to be the attacking water on the phosphate group, is not in the correct position for attack, probably an artefact due to the thiophosphate and calcium ions.

The consensus structures of the exonuclease active site exhibit a degree of DNA base disorder, suggesting that the bases may not have settled into their correct positions yet. Through further sub-classification of particles focused on the active site, we managed to enhance the DNA density for the 2-bp mismatch exonuclease state structure (Figure 4A). The two divalent metal ions essential for catalysis, in this instance Ca^2+^ replaces Mg^2+^, are both clearly identified (Figure 4A). Notably, D368, a key residue for exonuclease activity, is in a position to coordinate both metal ions within the pocket (48).

A third coordinated metal ion in close proximity to the active site in RB69 DNA polymerase is not seen in the HSV-1 DNA polymerase, and only one of the two coordinating residues, D275 in RB69 and D524 in HSV-1, is conserved between the two polymerases.

**Figure 5.**
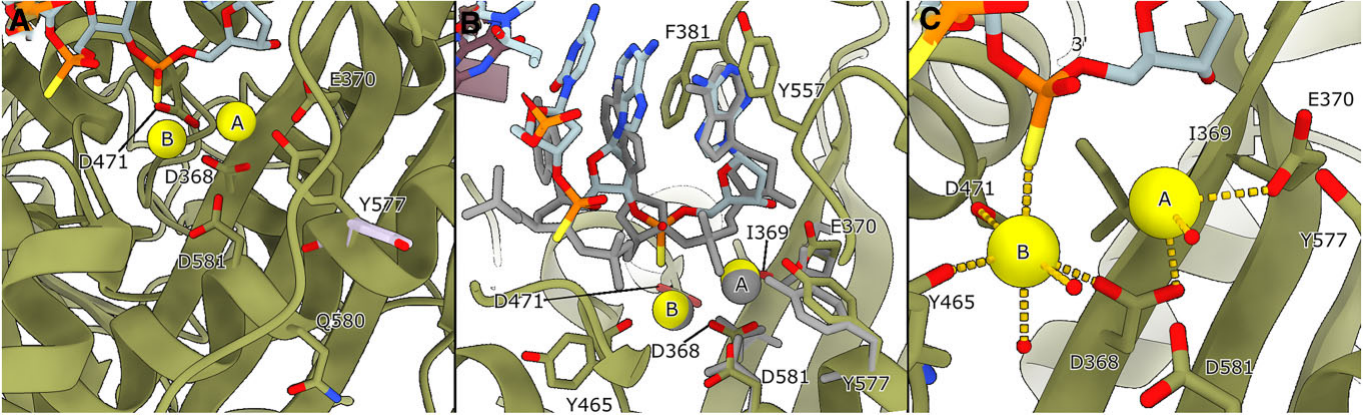
(**A**) The exonuclease active site of the 1-bp mismatch exonuclease state structure. Also in this structure the Ca^2+^ ions are easily identifiable. Y577 again has a double conformation, shown in green and plum. (**B**) The side chain conformations as well as the Ca^2+^ ion positions in the active site remain the same between the 1-bp mismatch (moss green) and 2-bp mismatch (grey) exonuclease state structures (the comparison is done with the highest resolution sub-state shown in Figure 4D). However, the DNA has shifted dramatically. The 3′-terminal base of the primer is in the same position and stacks with the side chain of Y557, but the thiophosphate group is now in a position to interact with metal B. (**C**) The coordination of the Ca^2+^ ions changes with the movement of the DNA, and metal B now directly interacts with the thiophosphate group. Metal A is lacking two of its coordination groups. One is potentially a water molecule located between the ions, but the map density is too diffuse to assign this unambiguously. A second water molecule is likely positioned between metal A and the DNA, replacing the coordination of the thiophosphate group present in the 2-bp mismatch exonuclease state structure. Still, also here the assignment is not unambiguous.

Mutations in the highly conserved Y577 (Y577F and Y577H) seem to severely or almost completely disrupt exonuclease activity (49,50), and its counterpart in *E. coli* DNA polymerase I (Y497) is directly involved in the catalytic reaction (51,52). A closer look at the gp43 structure from Escherichia phage RB69 (PDB-ID: 1CLQ) shows that the analogous tyrosine (Y323) faces outward (53). Interestingly, in our exonuclease state structures, the Y577 sidechain adopts two conformations: one outward-facing, akin to gp43, and another where it points towards the active site (Figures 4A and 5A). Moreover, Q580 exhibits dual conformations, with one reorienting towards the active site. Regrettably, the subclassification of particles could not segregate these conformations into distinct classes.

Through classification centred on the 2-bp mismatch exonuclease active site, we discerned two sub-states where several residues adopt different conformations. The DNA occupies its canonical position in the minor sub-state depicted in Figure 4B, but E370 and D581 do not. Metal B is coordinated between D368, D471, Y465’s main chain carbonyl, and two water molecules, one of which also interacts with the bridging 3′-oxygen atom. This contrasts with the mechanism proposed by Beese and Steitz, where metal B directly interacts with the 3′-oxygen atom (51,52). An additional ordered water is too far (3.3 Å) from metal B to help with coordination. Metal A, on the other hand, is coordinated by D368 and E370, along with D581 and the thiophosphate group in the DNA primer strand, possibly aided by a water molecule situated between the ion and I369 (Figure 4B, distances can be found in Supplementary Table S3). A second water molecule is likely implicated, bringing metal A to full coordination but it is not visible in this sub-state map.

Interestingly, we also identified a higher-resolution second sub-state where metal A shifts closer ‘underneath’ the thiophosphate group (Figure 4C and D, distances can be found in Supplementary Table S4). In this state, E370 and D581 rotate inward, and metal A achieves full coordination with the addition of the backbone carbonyl from I369, as well as the two proposed waters from the other sub-state, here visible. One of the waters, coordinated by Y577, is shifted compared to the water presumed to be forming a hydroxide ion in the canonical mechanism (51,52). In our structure, this water is coordinated by D581 instead of E370 (D501 and E357 in (51,52), respectively), placing it too far from the thiophosphate group to interact directly. The change from a phosphate to a thiophosphate group, in conjunction with the presence of calcium instead of magnesium ions, likely restricts the water from moving into the attacking position. The rotation of the Y577 side chain appears pivotal for the initial attack on the phosphate group via its interaction with the attacking hydroxide ion. Consequently, the thiophosphate group could also explain why Y577 does not fully adopt the inward-facing conformation. Metal B does not seem to shift between the different sub-states, but the final water comes into close enough proximity to achieve full coordination. A water molecule remains positioned between metal B and the bridging 3′-oxygen atom.

Across various classes, Q580 also displays considerable movement, though its precise significance remains uncertain.

The 1-bp mismatch active site closely resembles the exonuclease active site with the 2-bp mismatch DNA. The side chains adopt similar or identical orientations, and Y577 adopts a double conformation also in this structure (Figure 5A). The DNA in the exonuclease state active site is better resolved in the 1-bp mismatch structure as compared to the 2-bp mismatch structure. It is, however, shifted compared to its canonical position adopted by the 2-bp mismatch structure (Figure 5B). In Figure 5B, the 1-bp mismatch structure has been overlaid with the highest resolution structure of the 2-bp mismatch state (as shown in Figure 4D). The 3′-terminal base of the primer adopts a similar conformation and stacks with the Y557 side chain. The penultimate primer base also stacks with the side chain of F381 in this state. The coordination of the Ca^2+^ ions changes with the movement of the DNA. The thiophosphate group is now interacting directly with metal B instead of metal A as was shown for the 2-bp mismatch structure (Figure 5C). The coordination of metal B otherwise remains the same while metal A is lacking two of its coordination groups. Potentially a water molecule is located between the two metal ions but the map is too diffuse for unambiguous assignment. Likewise, a second water molecule is likely present between metal A and the DNA, but also here the assignment is ambiguous.

To test the editing fidelity in our system, we performed an *in vitro* DNA synthesis assay using a single mismatched nucleotide at the 3′ end of the primer. Strand-specific DNA sequencing of the newly synthesized DNA was determined by Sanger sequencing. The results showed a majority of our DNA synthesis products had undergone proofreading, thereby restoring complementarity to the template strand (Supplementary Table S2). The sequencing results, however, indicate a relatively large proportion of mismatch (∼25%) remaining in the final product. In support of this, the base substitution fidelity for HSV-1 UL30 has been shown to play a limited role in the overall fidelity and to depend on several factors, such as the mismatched pair and local sequence environment (48,54–55).

### The HSV-1 UL30–UL42 DNA interaction interface

**Figure 6.**
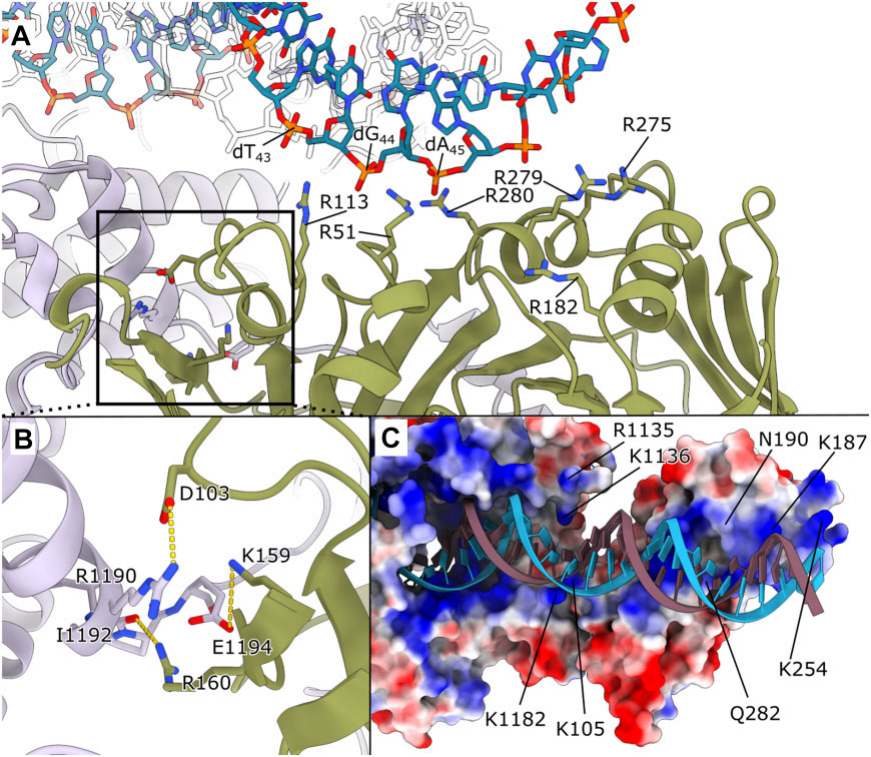
(**A**) Close-up view of the UL42 and DNA interaction site. The side chains that interact directly with the DNA backbone are R51, which interacts with the phosphate group of dG44 of the template strand; R113, which interacts with dT43; and R280, which interacts with dA45. R182, R275, and R279 are in position to take over the interactions following the rotation of DNA during elongation. (**B**) Potential salt bridges between UL30 and UL42. R1190 is in contact with D103, I1190 is in contact with R160, and Glu1194 is in contact with K159. (**C**) Electrostatic potential map of UL42, coloured by the local electrostatic potential (blue, +8 kT/e; red, −5 kT/e). The map displays interactions between the UL42 processivity factor and DNA, extending beyond the region modelled from our data unambiguously. Specifically, lysine residues K105, K187, and K254, as well as Q282 and N190 of UL42, are positioned to interact with DNA. In addition, R1135, K1136, and K1182 from the UL30 polymerase core might also interact, depending on the movement of the thumb region.

In previous crystal structures of UL30, the UL42 processivity factor was absent, leading to uncertainty about their interactions based on indirect information. Our structures demonstrate a remarkably flexible interaction where the primary interaction point is the C-terminus of UL30 (Figure 6A). This interaction functions as an anchor to keep UL42 tethered. Unlike the structural analogue PCNA, which forms a ring clamp, UL42 interacts more directly with the DNA. This anchoring arrangement allows for substantial flexibility and bending of the double-stranded DNA helix.

The interaction features three potential salt bridges: UL30 R1190 and UL42 D103, UL30 I1192 and UL42 R160, and UL30 E1194 and UL42 K159, which potentially contribute to interaction stabilisation (Figure 6B). However, not all these contacts are conserved across the various structures.

To explore the flexibility between domains and the DNA’s bending dynamics, we conducted a 3D flexible refinement (56). The resulting movies (Supplementary Movies S1 and S2) illustrate this flexibility, showcasing the back-and-forth and ‘up-and-down’ bending of the DNA. These structures offer direct evidence that the processivity factor functions as a DNA tether, as opposed to inducing a conformational shift in UL30 and/or UL42 to envelop the DNA, which is an alternative model previously suggested (57).

### The UL42 processivity factor-DNA interaction

In contrast to other DNA polymerase-associated processivity factors, like PCNA, which forms a ring-like clamp around DNA (53,58–59), UL42 is a DNA-binding protein. A comparison with the crystal structure of UL42 obtained in the absence of DNA confirms that DNA binding does not induce conformational changes (Supplementary Figure S6), a possibility previously discussed (25).

Zuccola *et al.* suggested a particular interaction site and side chains responsible for this interaction (R113, R182, R279, R280, and Q282) (25,60). We can here show that a number of both conserved arginines (R113, R182, R279, R280) and less conserved arginine R51 (not conserved among PRV, BHV, EHV) and R275 (L in PRV, K/R in BHV, EHV) play a pivotal role in interacting with the DNA strands (Figure 6A) (57). Additionally, K105 (not conserved), N190 (deletion in PRV, BHV, EHV), Q282 (K/R in PRV, BHV, EHV), and K254 could potentially interact with the DNA based on the electrostatic potential map (Figure 6C). Because of the inherent flexibility relative to UL30, the modelling of these potential side chains from our data is ambiguous, and we leave this as mere speculation.

The positively charged surface on UL42 extends onto UL30, forming a positively charged crevice for DNA interaction (Figure 6C). The conserved arginines all interact with the template DNA strand in our three models, and the orientation of the positively charged surface follows the helical pitch of the DNA backbone. This suggests that arginine residues likely move along the DNA backbone sequentially, with each arginine taking over the interaction as the DNA rotates. As a result, not all arginines interact with the DNA at the same time. Beyond the interactions described by Hayes et al. (22), we identified interactions with residue K1182 and potentially R1135 and K1136, located in a highly flexible region but positioned favourably for interaction.

## Concluding remarks

In conclusion, this study offers a comprehensive exploration of the structure of the DNA polymerase complex from Herpes Simplex Virus 1 (HSV-1) in various states of DNA replication. By capturing the complex in the pre-translocation state, the halted elongation state, and the DNA-editing state, we have gained a deeper understanding of the structural changes that underpin these critical processes. The pre-translocation state structure provides insights into the initial stages of the catalytic cycle, with the fingers domain in an open conformation, poised to accept incoming dNTPs. The halted elongation state structure showcases the intricate coordination between the polymerase and the DNA during active synthesis, notably how the fingers domain securely clasps the incoming dATP. Most importantly, the high-resolution DNA-editing state structure reveals the transition of the DNA from the polymerase to the exonuclease active site, facilitating proofreading and error correction. The varying conformations of the exonuclease active site, along with the engagement of metal ions, critical residues, and ordered water molecules, offer valuable insights into the enzymatic mechanisms at play. Moreover, the integration of the processivity factor within these structures offers a compelling depiction of its role in stabilizing the complex and its interactions with the DNA. The flexibilities observed in the interactions between the processivity factor, polymerase, and DNA underline the complex interplay between these components in guiding DNA replication and proofreading.

Collectively, these findings contribute significantly to our understanding of HSV-1 genome replication. By revealing the intricate molecular interactions underlying DNA synthesis, proofreading, and editing, our study not only advances fundamental knowledge of viral replication mechanisms but also lays a solid foundation for potential therapeutic interventions targeting HSV-1.

## Supplementary Material

gkae374_Supplemental_Files

## Data Availability

The coordinates have been deposited in the Protein Data Bank (PDB) with accession codes PDB-ID: 8OJ6 (Pre-translocation state structure), 8OJ7 (Halted elongation state structure), 8OJA (2-bp mismatch exonuclease state structure), 8OJB (2-bp mismatch exonuclease state active site), 8OJC (2-bp mismatch exonuclease state alternative active site), 8OJD (2-bp mismatch exonuclease state β-hairpin loop), 9ENP (1-bp mismatch exonuclease state structure), 9ENQ (1-bp mismatch exonuclease state active site). The cryo-EM maps have been deposited in the Electron Microscopy Data Bank (EMDB) with accession codes EMD-16906 (Composite map of pre-translocation state structure), EMD-16907 (Composite map of halted elongation state structure), EMD-16909 (Composite map of 2-bp mismatch exonuclease state structure), EMD-16910 (2-bp mismatch exonuclease state active site), EMD-16911 (2-bp mismatch exonuclease state alternative active site), EMD-16912 (2-bp mismatch exonuclease state β-hairpin loop), EMD-19837 (Composite map of 1-bp mismatch exonuclease state structure), EMD-19838 (1-bp mismatch exonuclease state active site), EMD-16918 (Focused refinement map of UL30 in pre-translocation state), EMD-16919 (Focused refinement map of UL42 in pre-translocation state), EMD-17014 (Consensus map of pre-translocation state), EMD-16924 (Focused refinement map of UL30 in halted elongation state), EMD-16925 (Focused refinement map of UL42 in halted elongation state), EMD-17013 (Consensus map of halted elongation state), EMD-16927 (Focused refinement map of UL30 in 2-bp mismatch exonuclease state), EMD-16928 (Focused refinement map of UL42 in 2-bp mismatch exonuclease state), EMD-17018 (Consensus map of 2-bp mismatch exonuclease state), EMD-19840 (Focused refinement map of UL30 in 1-bp mismatch exonuclease state), EMD-19841 (Focused refinement map of UL42 in 1-bp mismatch exonuclease state), EMD-19839 (Consensus map of 1-bp mismatch exonuclease state).
