# Supplementary material for: Dynamics of the Herpes simplex virus DNA polymerase holoenzyme during DNA synthesis and proof-reading revealed by Cryo-EM
Source: Nucleic Acids Res. 2024 May 29;52(12):7292–304. doi: 10.1093/nar/gkae374 (PMC11229320; doi:10.1093/nar/gkae374)
Supplement: gkae374_Supplemental_Files [file gkae374_supplemental_files.zip › Supplementary Information DNAp.pdf]

Supplementary Information -

Dynamics of the Herpes simplex virus DNA polymerase holoenzyme during DNA synthesis and proof-reading revealed by Cryo-EM

Emil Gustavsson[1,2], Kay Grünewald[2,3,4], Per Elias[5] and B. Martin Hällberg[1,2]*

[1] Department of Cell and Molecular Biology, Karolinska Institutet, 171 77 Stockholm, Sweden
[2] Centre for Structural Systems Biology, Deutsches Elektronen-Synchrotron DESY, Notkestraße 85, Building 15, 22607 Hamburg, Germany
[3] Leibniz-Institute of Virology, Martinistraße 52, 20251 Hamburg, Germany
[4] University of Hamburg, Department of Chemistry, Martin-Luther-King-Platz 6, 20146 Hamburg, Germany
[5] Institute of Biomedicine, Department of Medical Biochemistry and Cell Biology, Sahlgrenska Academy, University of Gothenburg, Box 440, 405 30 Gothenburg, Sweden

* To whom correspondence should be addressed. Tel: +46-704242094; Email: martin.hallberg@ki.se

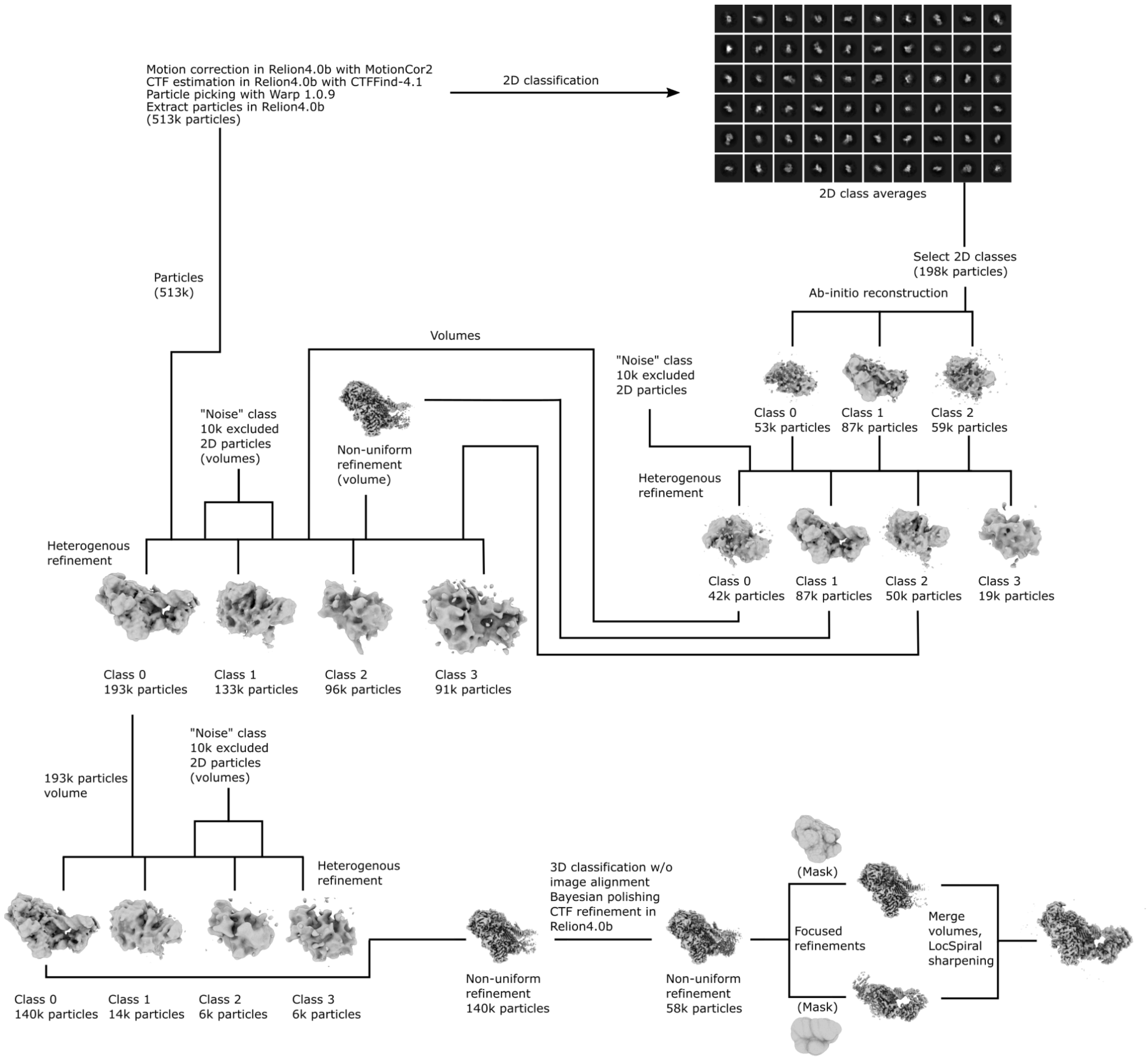

Supplementary Figure 1. Data processing scheme of DNA polymerase complex in the pre-translocation state. Motion correction and CTF estimation were performed in Relion 4.0b, with MotionCor2 and CTFFind 4.1, respectively (1–3). Particle picking was performed with Warp 1.0.9 (4), and extraction was performed in Relion 4.0b. The subsequent steps were performed in CryoSPARC v.3.2.0 (5), with the exception of Bayesian polishing and CTF refinement, which were done in Relion 4.0b.

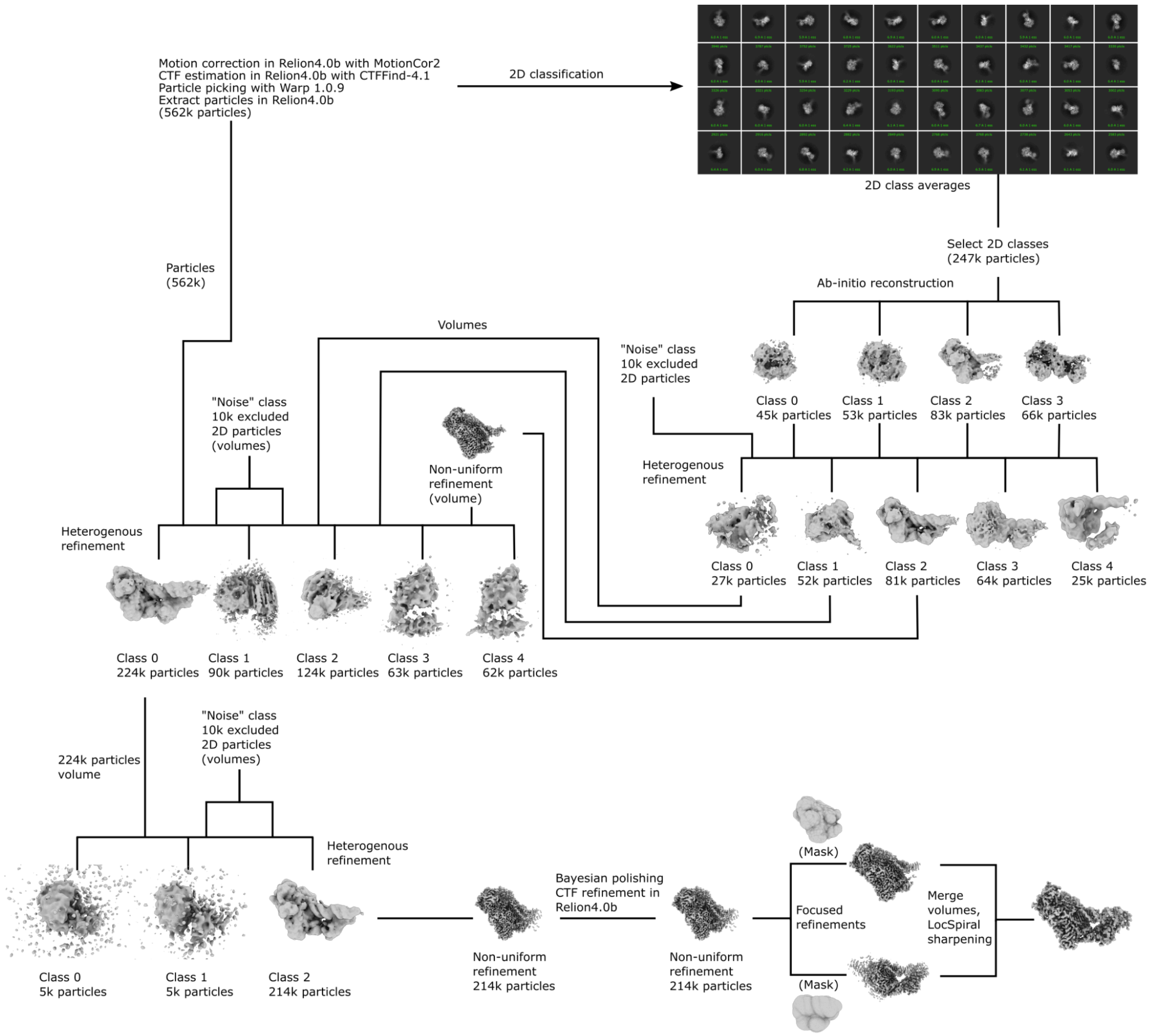

Supplementary Figure 2. Data processing scheme of DNA polymerase complex in the halted elongation state. Motion correction and CTF estimation were performed in Relion 4.0b, with MotionCor2 and CTFFind 4.1, respectively (1–3). Particle picking was performed with Warp 1.0.9 (4), and extraction was performed in Relion 4.0b. The subsequent steps were performed in CryoSPARC v.3.2.0 (5), with the exception of Bayesian polishing and CTF refinement, which were done in Relion 4.0b.

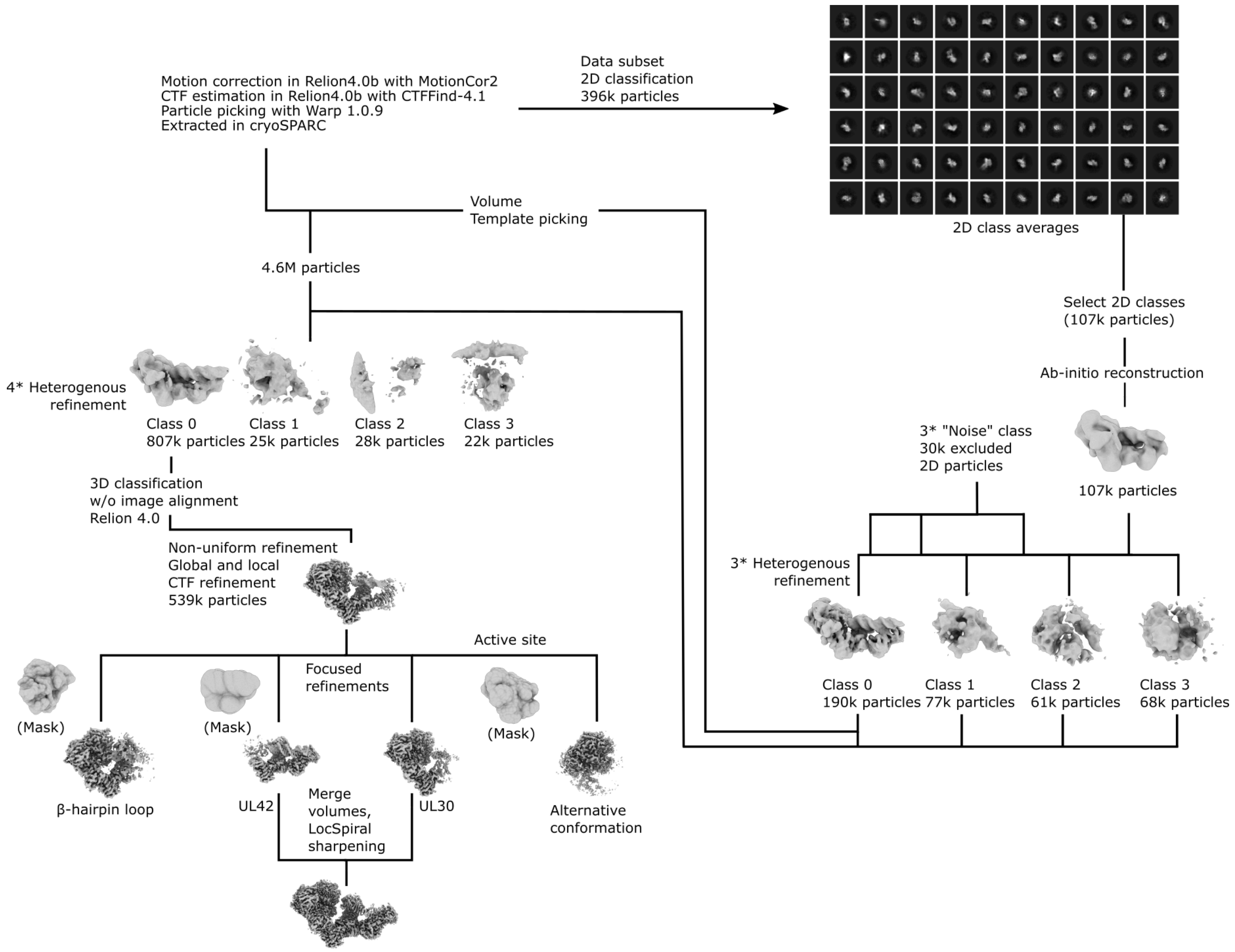

Supplementary Figure 3. Data processing scheme of DNA polymerase complex in the exonuclease state with 2-bp mismatch. Motion correction and CTF estimation were performed in Relion 4.0b, with MotionCor2 and CTFFind 4.1, respectively (1–3). Particle picking was performed with Warp 1.0.9 (4), and extraction was performed in Relion 4.0b. The subsequent steps were performed in CryoSPARC v.4.0.0 (5), with the exception of 3D classification, which was done in Relion 4.0b.

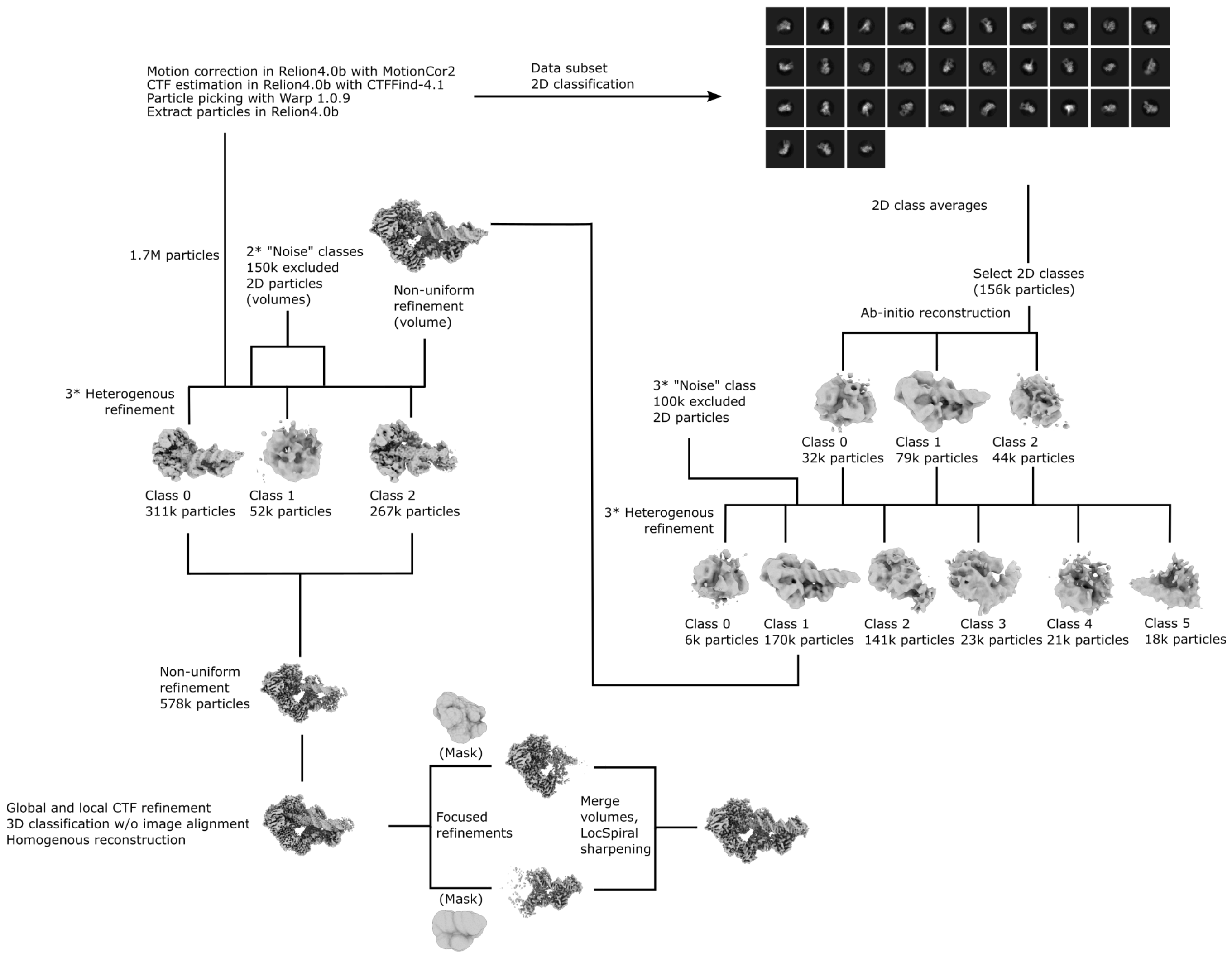

Supplementary Figure 4. Data processing scheme of DNA polymerase complex in exonuclease state with 1-bp mismatch. Motion correction and CTF estimation were performed in CryoSPARC v.4.4.1, with MotionCor2 and CTFFind 4.1, respectively (1–3). Particle picking was performed with Warp 1.0.9 (4). The subsequent steps were performed in CryoSPARC v.4.4.1 (5).

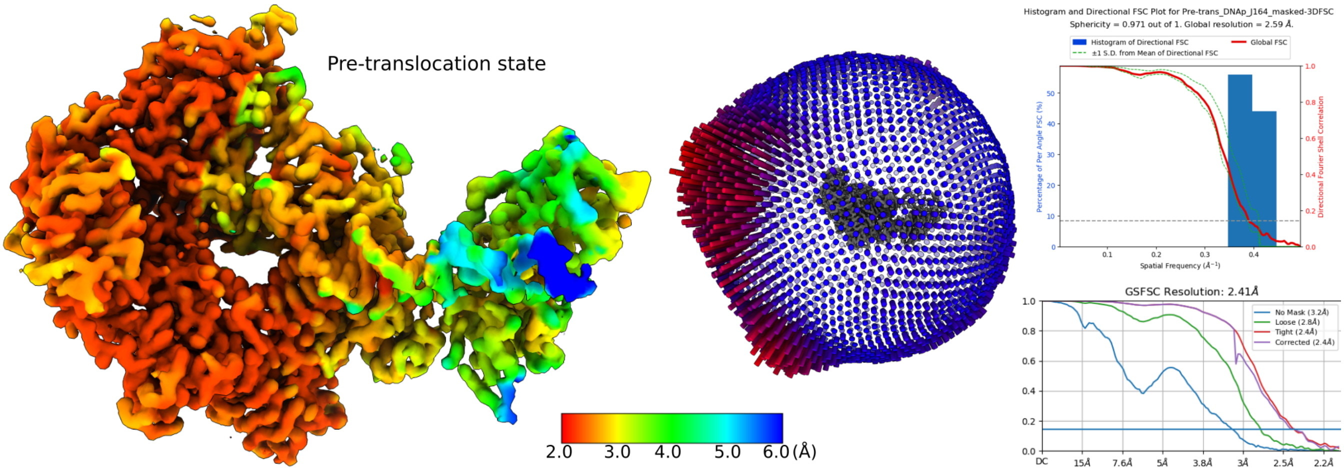

Supplementary Figure 5. CryoEM reconstruction post-processed by DeepEMhancer of the pre-translocated state colored by local resolution determined by local resolution estimation of the focused refinement maps in cryoSPARC (5, 6). The polymerase core reaches a resolution of 2.41Å, whereas the more peripheral domains, like the processivity factor and the end of DNA, are in the range of 3-4Å. There is a slightly preferred orientation, although not detrimental to the map reconstruction.

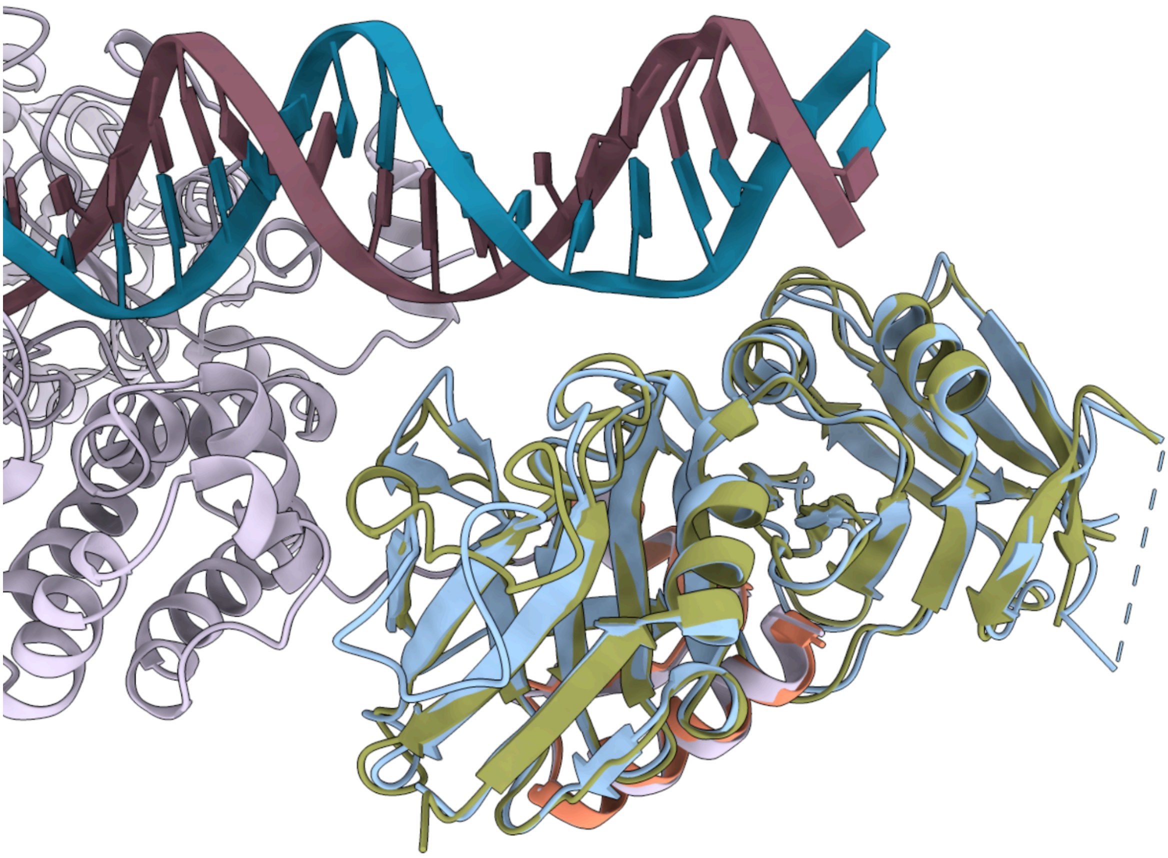

Supplementary Figure 6. Pre-translocation state structure of UL42 overlaid with the previously solved crystal structure (PDB-ID: 1DML, light blue, C-terminal peptide in orange)(7). No significant conformational change can be seen upon DNA binding.

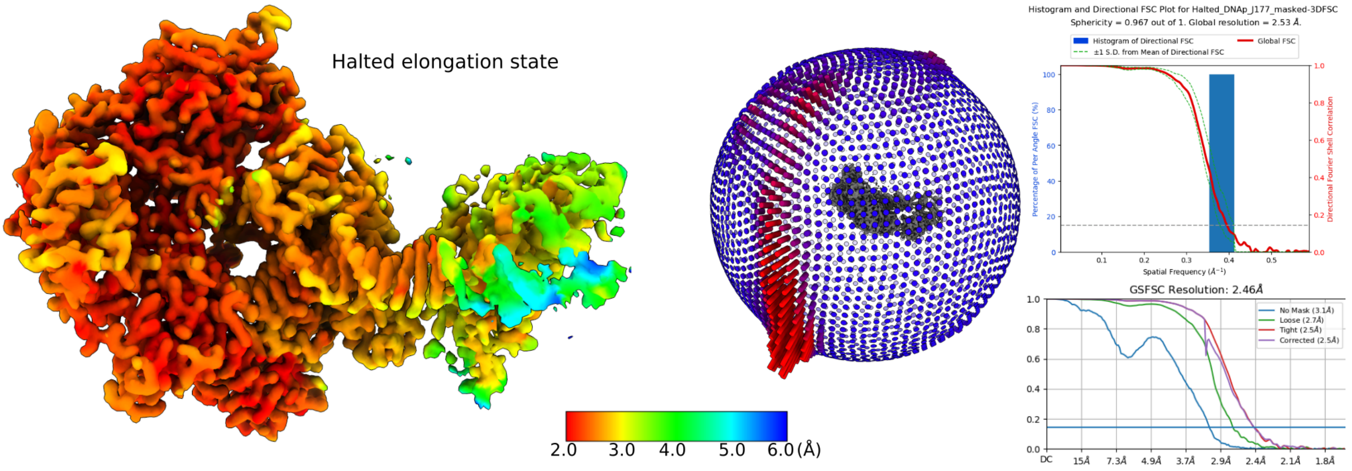

Supplementary Figure 7. Cryo-EM reconstruction post-processed by DeepEMhancer for the halted elongation state colored by local resolution determined by local resolution estimation of the focused refinement maps in cryoSPARC (5, 6). The polymerase core reaches a resolution of 2.46Å, whereas the more peripheral domains, like the processivity factor and the DNA end, are in the range of 3-4Å. There is a slightly preferred orientation, although not detrimental to the map reconstruction.

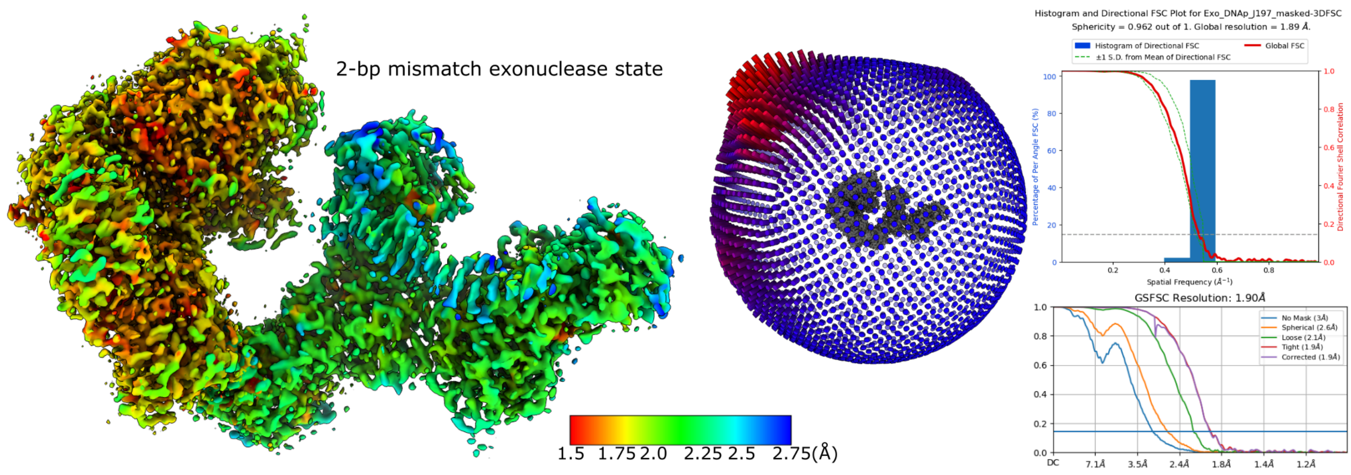

Supplementary Figure 8. Locally filtered cryo-EM reconstruction of the 2-bp mismatch exonuclease state colored by local resolution determined by local resolution estimation of the focused refinement maps in cryoSPARC (5). The polymerase core reaches a resolution of 1.90Å, whereas the more peripheral domains like the processivity factor and the end of DNA are in the range of 2-2.75Å. The DNA itself is poorly resolved due to flexibility, but is visible at lower contour levels. There is a slightly preferred orientation, although not detrimental to the map reconstruction.

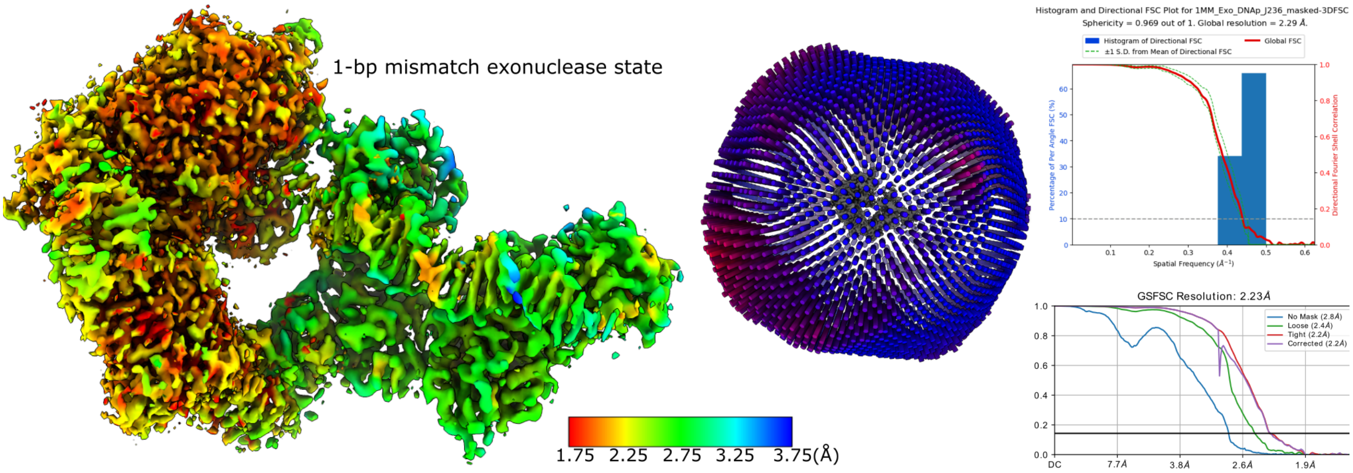

Supplementary Figure 9. Locally filtered cryo-EM reconstruction of the 1-bp mismatch exonuclease state colored by local resolution determined by local resolution estimation of the focused refinement maps in cryoSPARC (5). The polymerase core reaches a resolution of 2.1Å, whereas the more peripheral domains like the processivity factor and the end of DNA are in the range of 2-2.75Å. The DNA itself is poorly resolved due to flexibility, but is visible at lower contour levels. The particles have a more even orientation distribution compared to the other structures.

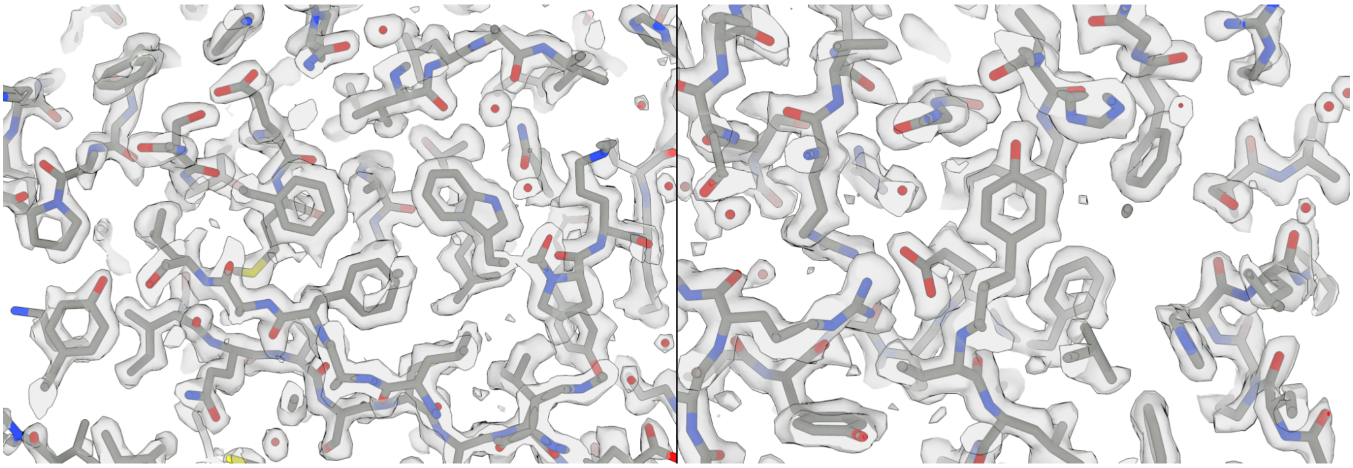

Supplementary Figure 10. Aromatic residues in the exonuclease state of UL30 show clear rings, and water molecules can be clearly identified (2-bp mismatch exonuclease structure).

Supplementary Table 1. Cryo-EM data collection, refinement and validation statistics. This table provides the parameters and statistics for the data collection, processing, refinement, and structure validation of the cryo-EM structures. Refinement statistics were generated using the Servalcat package.

	**Pre-translocation**	**Halted elongation**	**Exonuclease state 2bp mismatch**	**Exonuclease state 2bp mismatch active site**	**Exonuclease state 2bp mismatch alternative site**	**Exonuclease state 2bp mismatch beta-hairpin loop**	**Exonuclease state 1bp mismatch**	**Exonuclease state 1bp mismatch active site**
**PDB code**	8OJ6	8OJ7	8OJA	8OJB	8OJC	8OJD	9ENP	9ENQ
**EMDB code**	Composite: EMD-16906<br>Full: EMD-17014<br>Focused UL30: EMD-16918<br>Focused UL42: EMD-16919	Composite: EMD-16907<br>Full: EMD-17013<br>Focused UL30: EMD-16924<br>Focused UL42: EMD-16925	Composite: EMD-16909<br>Full: EMD-17018<br>Focused UL30: EMD-16928<br>Focused UL42: EMD-16927	EMD-16910	EMD-16911	EMD-16912	Composite: EMD-19837<br>Full: EMD-19839<br>Focused UL30: EMD-19840<br>Focused UL42: EMD-19841	EMD-19838
**Data collection and processing**								
Microscope/detector	Krios G3i/K3	Krios G3/K3	Krios G3i E-CFEG/K3				Krios G3i E-CFEG/K3	
Magnification	165kx	105kx	165kx				165kx	
Voltage	300kV	300kV	300kV				300kV	
Electron exposure (e-/Å^2)	58	50	58				58	
Defocus range (μm)	-0.5 to -1.5	-0.5 to -2	-0.5 to -1.5				-0.3 to -1.5	
Pixel size (Å)	0.505	0.85	0.505				0.5076	
Symmetry imposed	C1	C1	C1				C1	
Final	58332	164520	539028	142948	48633	41759	577658	163943

particle images (*n*)								
Reconstruction method	Single particle reconstruction	Single particle reconstruction	Single particle reconstruction	Single particle reconstruction	Single particle reconstruction	Single particle reconstruction	Single particle reconstruction	Single particle reconstruction
Map resolution (Å) ($FSC_{0.143}$)	2.4	2.5	1.9	1.9	2.1	2.5	2.2	2.1
Map sharpening B factor (Å^2)	Local Filter	Local Filter	Local Filter	Local Filter	Local Filter	Local Filter	Local Filter	Local Filter
**Refinement**								
Initial model	7LUF, 1DML	7LUF, 1DML	7LUF, 1DML	7LUF	7LUF	7LUF	7LUF, 1DML	7LUF
**Model composition**								
Non-hydrogen atoms	11650	12026	11782	6785	3501	8674	11552	5743
Protein residues	Chain A: 1085 Chain B: 266	Chain A: 1089 Chain B: 266	Chain A: 1072 Chain B: 266	Chain A: 835	Chain A: 395	Chain A: 976	Chain A: 1072 Chain B: 266	Chain A: 707
Nucleotide residues	Chain C: 22 Chain D: 29	Chain C: 29 Chain D: 38	Chain C: 29 Chain D: 24	Chain C: 3	Chain C: 3	Chain C: 23 Chain D: 23	Chain C: 25 Chain D: 24	Chain C: 3 Chain D: 2
Water	4	-	176	116	288	-	7	7
Other	2 Mg^{2+}	2 Mg^{2+} 1 dATP	2 Ca^{2+}	2 Ca^{2+}	2 Ca^{2+}	1 Ca^{2+}	2 Ca^{2+}	2 Ca^{2+}
**R.m.s. deviations (RMSZ)**								
Bond lengths	0.46	0.50	0.40	0.43	0.44	0.42	0.41	0.37
Bond angles	0.74	0.80	0.73	0.72	0.69	0.77	0.80	0.71
**Validation**								
MolProbity score	1.46	1.39	1.42	0.98	1.12	1.45	1.04	0.86
Clashsco	5.20	4.05	4.88	1.20	3.31	3.37	2.52	1.32

re								
Poor rotamers (%)	0	0	0	1	1	1	1	0
**Ramachandran plot**								
Favored (%)	97	97	97	97	98	95	98	99
Allowed (%)	3	3	3	3	2	5	2	1
Disallowed (%)	0	0	0	0	0	0	0	0

Supplementary Table 2. Sanger sequencing results of proofreading reaction. The mismatch introduced (position 39) is highlighted in bold.

Position	Base Call	Quality Score	A Value	C Value	G Value	T Value
1	C	7	0	0	0	0
2	C	7	0	0	0	0
3	C	7	5	9	0	0
4	G	5	21	3	46	5
5	A	14	19	1	4	0
6	G	15	0	8	26	0
7	A	24	30	0	0	0
8	T	12	36	0	0	43
9	T	22	0	0	0	58
10	T	45	0	0	0	88
11	G	27	0	0	285	0
12	C	34	0	61	40	0
13	T	27	0	0	16	116
14	G	28	1	0	114	0
15	A	27	423	45	0	0
16	C	38	10	261	0	0
17	T	49	0	3	0	320
18	T	50	0	0	0	411
19	T	28	0	0	60	360
20	G	41	0	0	961	11
21	T	51	0	0	1	314
22	T	58	0	2	0	486
23	C	46	0	445	0	31
24	T	46	70	2	0	371
25	A	26	314	0	0	123
26	A	46	523	0	0	0
27	T	61	0	0	0	614
28	T	61	0	0	2	878
29	G	58	0	0	993	0
30	A	28	668	0	2	0
31	G	61	19	0	712	0
32	T	61	0	0	1	791
33	T	58	0	0	8	960
34	G	58	0	0	1687	0
35	G	58	0	0	1093	0
36	T	60	0	0	2	728
37	T	57	0	0	10	1114
38	G	16	0	0	2232	1
**39**	**G**	**10**	**0**	**0**	**444**	**141**
40	A	24	752	41	2	1
41	C	27	105	848	5	0
42	G	46	0	2	991	0

43	G	58	0	1	1820	0
44	C	52	0	672	0	1
45	T	52	0	1	6	1377
46	G	46	0	1	1337	3
47	C	38	0	1054	1	0
48	G	38	0	6	701	0
49	A	29	1187	0	4	0
50	G	29	106	0	673	0
51	G	51	0	1	1832	0
52	C	51	0	774	5	0
53	G	38	1	0	1156	0
54	A	36	902	0	1	2
55	T	35	72	1	0	1249
56	C	38	14	1191	0	2
57	A	27	733	0	7	0
58	G	45	1	0	1068	0
59	G	45	0	0	2730	2
60	T	46	0	0	41	789
61	G	44	0	0	2362	1
62	T	48	0	1	1	888
63	C	46	0	1363	1	1
64	G	42	0	1	754	1
65	T	39	4	0	1	681
66	A	28	1520	0	50	1
67	G	26	211	0	921	1
68	T	9	232	0	156	899
69	G	16	174	0	1012	2
70	A	8	1548	0	14	0
71	T	3	112	0	0	10
72	A	7	68	0	0	1
73	A	11	1	1	0	3
74	T	16	0	0	0	11
75	T	11	0	0	0	4
76	T	10	0	0	0	3
77	C	5	0	0	7	0
78	G	5	0	0	8	0
79	T	5	0	0	6	2
80	G	5	0	0	4	0
81	G	5	0	0	8	0
82	G	3	0	0	3	0
83	G	5	1	0	17	0
84	G	4	0	0	3	0
85	A	3	6	2	4	0
86	T	3	0	0	2	5
87	C	8	2	4	0	0
88	C	8	2	3	0	2

89	C	10	1	5	0	0
90	C	3	1	3	1	0
91	A	3	2	3	0	0
92	C	3	1	3	0	0
93	G	3	2	0	4	0
94	G	5	1	2	3	0
95	A	4	5	0	0	0
96	C	5	1	2	2	1
97	T	9	5	0	0	6
98	G	9	3	0	5	0

Supplementary Table 3. Coordinating distances in 2-bp mismatch exonuclease active site, less populated class (Figure 4B in main paper).

**Metal ion**	**Residue**	**Distance (Å)**
Metal A	Tyr465	2.5
Metal A	Asp368	2.4
Metal A	Asp471 contact 1	2.4
Metal A	Asp471 contact 2	2.5
Metal A	Water	2.3
Metal A	Water	2.7
Metal B	DA (thiophosphate)	2.8
Metal B	Asp581	2.4
Metal B	Asp368	2.4
Metal B	Glu370	2.4
Metal B	Ile369	4.4
Metal B	-	-

Supplementary Table 4. Coordinating distances in 2-bp mismatch exonuclease active site, class with highest resolution (Figure 4C and D in main paper).

**Metal ion**	**Residue**	**Distance (Å)**
Metal A	Tyr465	2.5
Metal A	Asp368	2.5
Metal A	Asp471	2.5
Metal A	Water	2.1
Metal A	Water	2.6
Metal A	Water	2.9
Metal B	DA (thiophosphate)	2.9
Metal B	Asp368	2.4
Metal B	Ile369	2.4
Metal B	Glu370	2.4
Metal B	Water	2.5
Metal B	Water	3.2

Supplementary Movie 1. Movie from 3D flexible refinement in cryoSPARC of the movement of the processivity factor (moss green) and DNA relative to the polymerase core (plum) in the halted DNA polymerase dataset. The processivity factor moves together with the DNA “up-and-down” and “side-to-side” (8).

Supplementary Movie 2. Movie of the halted elongation state structure morphed between frames 1, 21 and 41 of Supplementary Movie 1.

Supplementary references

1. Zivanov,J., Nakane,T. and Scheres,S.H.W. (2020) Estimation of high-order aberrations and anisotropic magnification from cryo-EM data sets in *RELION* -3.1. *IUCrJ*, **7**, 253–267.
2. Zheng,S.Q., Palovcak,E., Armache,J.-P., Verba,K.A., Cheng,Y. and Agard,D.A. (2017) MotionCor2: anisotropic correction of beam-induced motion for improved cryo-electron microscopy. *Nat. Methods*, **14**, 331–332.
3. Rohou,A. and Grigorieff,N. (2015) CTFFIND4: Fast and accurate defocus estimation from electron micrographs. *J. Struct. Biol.*, **192**, 216–221.
4. Tegunov,D. and Cramer,P. (2019) Real-time cryo-electron microscopy data preprocessing with Warp. *Nat. Methods*, **16**, 1146–1152.
5. Punjani,A., Rubinstein,J.L., Fleet,D.J. and Brubaker,M.A. (2017) cryoSPARC: algorithms for rapid unsupervised cryo-EM structure determination. *Nat. Methods*, **14**, 290–296.
6. Sanchez-Garcia,R., Gomez-Blanco,J., Cuervo,A., Carazo,J.M., Sorzano,C.O.S. and Vargas,J. (2021) DeepEMhancer: a deep learning solution for cryo-EM volume post-processing. *Commun. Biol.*, **4**, 874.
7. Zuccola,H.J., Filman,D.J., Coen,D.M. and Hogle,J.M. (2000) The Crystal Structure of an Unusual Processivity Factor, Herpes Simplex Virus UL42, Bound to the C Terminus of Its Cognate Polymerase. *Mol. Cell*, **5**, 267–278.
8. Punjani,A. and Fleet,D. (2022) 3D Flexible Refinement: Structure and Motion of Flexible Proteins from Cryo-EM. *Microsc. Microanal.*, **28**, 1218.
